# Eating Attitudes of Patients with Celiac Disease in Brazil: A Nationwide Assessment with the EAT-26 Instrument

**DOI:** 10.3390/nu15224796

**Published:** 2023-11-16

**Authors:** Luiza Franco, Eduardo Yoshio Nakano, António Raposo, Hmidan A. Alturki, Sehad N. Alarifi, Cláudia Chaves, Edite Teixeira-Lemos, Bernardo Romão

**Affiliations:** 1 Instituto de Educação Superior de Brasilia, IESB University Center, Brasília 70200-730, Brazil; luizabfranco@gmail.com; 2 Department of Statistics, University of Brasília (UnB), Brasília 70910-900, Brazil; eynakano@gmail.com; 3 CBIOS (Research Center for Biosciences and Health Technologies), Universidade Lusófona de Humanidades e Tecnologias, Campo Grande 376, 1749-024 Lisboa, Portugal; 4 General Directorate for Funds & Grants, King Abdulaziz City for Science & Technology, Riyadh 11442, Saudi Arabia; halturki@kacst.edu.sa; 5 Department of Food and Nutrition Science, Al-Quwayiyah College of Sciences and Humanities, Shaqra University, Shaqraa 11971, Saudi Arabia; snalarifi@su.edu.sa; 6 ESSV, Centre for Studies in Education and Innovation (CI&DEI), Polytechnic University of Viseu, 3504-510 Viseu, Portugal; claudiachaves21@gmail.com; 7 CERNAS Research Centre, Polytechnic University of Viseu, 3504-510 Viseu, Portugal; etlemos3@gmail.com

**Keywords:** Brazil, celiac disease, eating attitudes, gluten-free diet

## Abstract

Celiac disease (CD) is an immune-mediated enteropathy triggered by the ingestion of gluten in genetically predisposed individuals. In this sense, a gluten-free diet is the only safe treatment available. Due to the restrictions resulting from this eating pattern, this treatment may impair the relationship of the people with CD with food, increasing the risk of a disordered eating attitude, which is associated with eating disorders. The EAT-26 is a validated instrument already applied worldwide in different populations, and higher scores are suggestive of eating attitudes prone to evolve into eating disorders. Studies carried out in other countries have already shown that people with CD are prone to developing eating disorders; however, no study has been carried out with this theme in the population with CD in Brazil. We carried out a nationwide cross-sectional study in three steps: (i) study design and instrument; (ii) recruitment of participants and ethics; (iii) statistical analysis. A total of 385 participants were included in our sample, 96.36% of them being women. The internal consistency of the applied self-administered Brazilian version of the EAT-26 online questionnaire presented a satisfactory Cronbach’s alpha of 0.812, and in total, 36.1% of the respondents were classified with a disordered eating attitude. No differences were found among the scores of participants when divided by categories regarding gender, average monthly income, age, and educational level. However, scores classified as a disordered eating attitude were found in respondents with a body mass index classified as overweight and obese. Our study highlights that disordered eating attitudes are present in overweight and obese women with celiac disease; thus, public health politics are needed to prevent and treat these attitudes.

## 1. Introduction

Celiac disease (CD) is an immune-mediated enteropathy triggered by the ingestion of gluten (main protein fraction present in wheat, rye, and barley) in genetically predisposed individuals [1,2,3,4]. As for its distribution around the world, studies demonstrate that serological and histological prevalence rates worldwide for the disease are 1.4% and 0.7%, respectively [5]. Clinical signs are broad and may contain typical intestinal features, such as chronic diarrhea, weight loss, and abdominal distention, and atypical recurrent abdominal pain, aphthous stomatitis, short stature, high levels of aminotransferase, fatigue chronic, and reduced bone mineral density, including an iron deficiency with or without anemia [2,6,7,8,9]. In addition, CD is associated with an increased prevalence of lymphoproliferative disease, infertility, cancer, and risk of fractures [10,11,12,13].

Currently, despite the presence of gluten-specific digestive enzymes on the market, the only safe treatment is the gluten-free diet (GFD); therefore, foods containing this protein fraction should be totally excluded from the diet [1].

The GFD is characterized as a challenging treatment since, in addition to the presence of gluten in traditional food of the Brazilian diet, there is also the risk of the accidental ingestion of this protein fraction through cross-contamination [14,15]. Furthermore, it appears that the cost of these products is higher, and their availability is reduced when compared to their equivalent counterparts that contain gluten [16,17,18].

Therefore, due to the restrictions arising from this dietary pattern, this treatment may impair the relationship of people with CD with food, increasing the risk of eating disorders [19,20].

Eating disorders (ED) are characterized as abnormal attitudes related to eating or weight control, have a multifactorial etiology, and can be deadly while also considerably impairing the individual’s physical health and psychosocial functioning [21,22,23]. There are several diagnostic methods, such as DSM-5 and ICD-11 [24,25] as well as those that assess attitudes associated with their development, such as the Binge Eating Scale (ECAP), Edinburgh Bulimic Investigation Test (BITE), Restraint Scale, Hay Questionnaire, Eating Attitudes Scale (DEAS), and the Eating Attitudes Test (EAT-26) [26].

EAT-26, in turn, is a 26-item validated instrument that assesses eating attitudes associated with the development of eating disorders, stating that results above the cutoff point suggest higher risk for developing ED [27]. As it is a short, easy-to-apply instrument, it is consolidated as a screening tool for assessing the risk of developing eating disorders in at-risk populations [27].

Studies carried out in other countries have already shown that people with CD are prone to developing EDs. In Italy, a 2013 study indicate that the frequency of altered eating attitude is increased in untreated CD when compared to a control group, with percentage of pathological EAT-26 scores significantly different between groups [28]. In Israel, a 2018 study in adolescents revealed that individuals with CD obtained higher scores on the topics diet, body image, and concern about food [29]. In addition, in the United Kingdom, in 2016, a study showed a higher prevalence of eating disorders assessed with the EAT-26 in celiac disease compared to a healthy control group, with a score of 15.7% above the clinical cutoff point [19].

In this sense, studies regarding dysfunctional eating attitudes among people with CD are needed given that these comorbidities are directly associated with the overall quality of life and the long-term adoption of a GFD [30,31]

However, to date, no study has been carried out in the CD population in Brazil. Therefore, the objective of this study is to analyze the eating attitudes of individuals with celiac disease and relate them to the indication of eating disorders.

## 2. Materials and Methods

### 2.1. Study Design and Instrument

**Figure 1:**
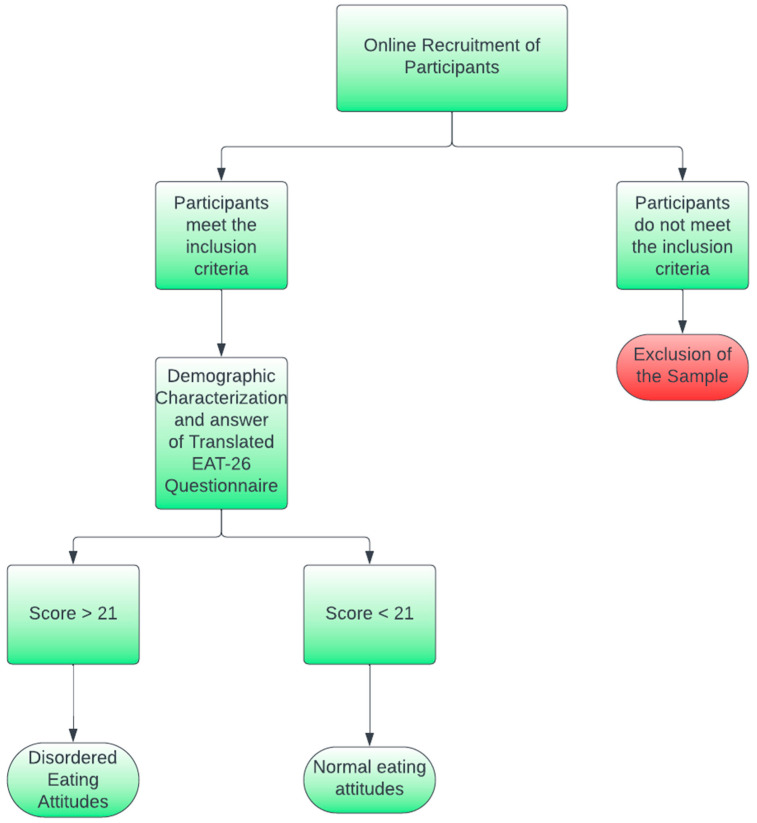
Schematic diagram of the study design.

A nationwide quantitative cross-sectional study regarding the eating attitudes of patients with CD in Brazil was performed using a validated self-administered instrument (EAT-26), translated to Brazilian Portuguese, and validated [27,32]. A schematic diagram of the study design is available in Figure 1 below.

The EAT-26 questionnaire aims to screen disordered eating attitudes associated with eating disorders and comprises 26 items, with its possible answers distributed among a 6-item Likert-type scale (Always, Usually, Often, Sometimes, Rarely, and Never) [27,32]. Each question is evaluated utilizing a 5-point scale, with “Always” corresponding to 5 points; “Often”, 3 points; and “Never”, 0 points. In this sense, the maximum punctuation for the questionnaire is 130.

According to the instrument, participants with scores equal or above 21 were classified with disordered eating attitudes [27,32]. As a part of the EAT-26 questionnaire, self-reported weight and height were also collected, and the respective Body Mass Index (BMI) (kg/m^2^) was calculated and classified for each participant according to World Health Organization (WHO) guidelines [33]. The English version of the EAT-26 questionnaire is available in Appendix A.

Besides the Brazilian version of the EAT-26, sociodemographic characteristics previously defined by the Brazilian Institute of Geography and Statistics (IBGE; Instituto Brasileiro de Geografia e Estatística) were also collected, such as gender, place of residency in Brazil, age, average monthly income (BRL), and educational level. For better comprehension of the average monthly income, BRL was converted to USD, utilizing the conversion scale of 1 USD = 4.81 BRL. Google Forms^®^ was used to collect answers of Brazilian patients with CD from 1 May to 1 June 2023.

### 2.2. Recruitment of Participants and Ethics

The inclusion criteria of participants were (i) to have celiac disease diagnoses for at least two years; (ii) to live in Brazil; (iii) to be at least 18 years of age. Those who did not agree to participate on the research were directed to a page appreciating their time.

The study was approved by the ethics committee of University Center IESB (Instituto de Educação Superior de Brasília) with the registry number CAAE: 69334823.0.0000.8927 and was also conducted according to the Declaration of Helsinki guidelines.

Participants were recruited through artwork posted on social media networks, such as Instagram^®^, Facebook^®^, Whatsapp^®^, and Tik Tok^®^. In order to obtain a more probative sample of Brazilians with celiac disease, a partnership was signed with the National Federation of Celiac Disease of Brazil (FENACELBRA) and its respective affiliated associations distributed among twenty-six Brazilian states and Federal District (ACELBRAS). Given the low prevalence of celiac disease, a convenience sample was used to determine the number of participants.

### 2.3. Statistical Analysis

As a resource of Google Forms^®^ platform, all items were mandatory to be completed; in this sense, no missing answers were present. Regarding the internal consistency of the questionnaire, the Cronbach’s alpha was calculated with a level of significance of 95% (CI 95%), noting that results equal or above of 0.7 were interpreted as a reliable internal consistency.

Quantitative statistics were presented as their mean and standard deviation. The student’s *t*-test and one-way analysis of variance (ANOVA) followed by Tukey’s post-hoc test with a significance level of 95% (*p* < 0.05) were performed in order to compare average scores presented by the EAT-26 questionnaire in different stratums, such as BMI (kg/m^2^), gender, age, monthly income, and educational level. Software tools Microsoft Excel^®^ (United States, 2023) and IBM SPSS Statistics for Windows (IBM Corp., Armonk, NY, USA, 2023) were used to perform all analyses.

Afterwards, the items were divided according to the EAT-26 scales: Diet Scale (D), Bulimia and Food Preoccupation Scale (B), and Oral Control Scale (OC), then analysis of variance (ANOVA, *p* < 0.05) was applied to statistically compare the scales.

As for the individual analysis of the items in the instrument, first, the total percentages of the answers for each item were grouped. Then, the answers for each item were expressed as percentages of their frequencies. A comparison based on the absolute frequencies between answers of respondents classified with a normal eating attitude and a disordered eating attitude was performed.

## 3. Results

### 3.1. Characteristics of the Included Participants

Data collected with the online self-administration of the EAT-26 among Brazilian individuals with CD presented a total response of 394 individuals; however, only 385 participants met the inclusion criteria (>18 years of age), representing in this manner the final sample of the study. The majority of respondents were female (96.36%; *n* = 371), while only 3.37% (*n* = 13) were men. Only one person declared themselves as non-binary (0.25%). A full description of the sociodemographic and BMI data regarding this study’s participants is available in Table 1.

Overall, most respondents (*n* = 112; 39.09%) presented an average monthly income of ranging from 5000.01 BRL (USD 1038.71) to 10,000.01 BRL (USD 2077.43), while the minor parcel of respondents (*n* = 76; 19.74%) presented an income of up to 3000.00 BRL (USD 623.00).

Regarding the average age of the respondents, most of our sample comprised people ranging between 25 and 34 years of age (*n* = 136; 35.32%), with people ranging between 18 and 24 years representing the second higher number of participants (*n* = 110; 28.57%). The lower number of participants were above 55 years of age (*n* = 2; 5.19%).

As for the educational level, almost half of the respondents were at the undergraduate level (*n* = 189; 49.09%), while 141 respondents (29.61%) informed us of being at a graduate level or above. Only 14.28% (*n* = 55) of the sample studied until high school only.

Regarding the average BMI of respondents, which was calculated with self-reported weight and height collected through the online questionnaire, most of the sample (*n* = 231; 60%) were classified as normal weight, presenting an average BMI of 17.23 ± 9.13, followed by 21.81% of the sample being classified as overweight (21.44 ± 10.09 Kg/m^2^).

**Figure 2:**
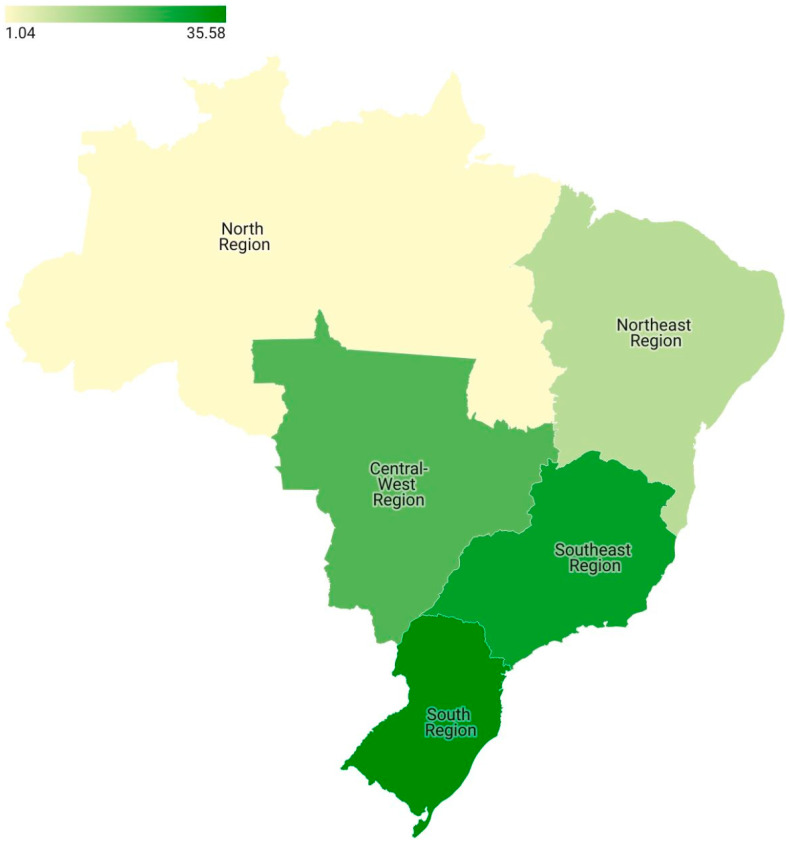
Coropletic map generated from the place of living of respondents in the Brazilian five regions.

A distribution of the place of living among respondents on the five regions of Brazil is disposed as a coropletic map in Figure 2.

In general, most respondents were from the South region (*n* = 137; 35.85%), followed by 115 respondents residing in the Southeast region (29.87%). The Central–West region contributed 22.86% of the respondents (*n* = 88), while the Northeast and North regions contributed 10.65% (*n* = 41) and 1.04% (*n* = 4), respectively.

### 3.2. Questionnaire Internal Consistency and Obtained Scores

Among all respondents, 36.1% (*n* = 139) scored above the cutoff points (>21), resulting in the classification as patients with disordered eating attitudes. Regarding the Cronbach’s alpha calculated to evaluate the internal consistency of the questionnaire, the average score of the questionnaire was 19.06 ± 10.06, and the alpha was 0.812 (CI 95%: 0.784–0.838), resulting in a satisfactory internal consistency (>0.7).

As for the average score collected through the online administration of the EAT-26 questionnaire, the scores stratified by sociodemographic variables and average BMI are available in Table 2 below.

Regarding the gender variable, female respondents presented an average score of 19.25 ± 10.05 points, a higher score in comparison to what is found among male respondents (11.85 ± 3.59). However, none of the groups presented an average score classified as a disordered eating attitude.

As for the average monthly income, no differences were found between all groups of respondents. In addition, no differences were found between respondents among different ages. The educational level seemed to be a determinant factor regarding the obtained scores. Respondents who studied up until high school (20.02 ± 10.27) and obtained an undergraduate degree (20.14 ± 10.47) presented significantly higher scores in comparison to respondents with a graduate degree (17.23 ± 9.18); however, none of the groups were classified with a disordered eating attitude.

Respondents classified both with underweight and adequate weight BMIs did not present significant differences between the obtained scores and did not present a score associated with a disordered eating attitude; nevertheless, overweight and obese respondents presented scores classified as a disordered eating attitude (>21), differing from other groups and presenting higher scores.

The analysis of the questionnaire scales showed that there is no statistically significant difference between their means (*p* > 0.05). This means that we cannot conclude that there are significant differences in respondent scores between the questionnaire’s scales.

However, regarding the individual items of the instrument, between the groups with normal (Score < 21) and disordered (Score > 21) eating attitudes, different frequencies for each item were found. Table 3 below presents the frequencies of the answers per item as percentages. A full description of each item is available in Appendix A. Also, the full answer sheet for the collected data is available at Table S1 (Supplementary file).

In general, answers marked as “Always”, “Usually”, and “Often” are indicative of disordered eating attitudes. As an example, item 1, related to the fear of weight gain due to food intake, was more frequent in the group with scores above 21, and in all items, with the exception of item 19, it was described as “I demonstrate self-control in relation to foods” (Appendix A).

## 4. Discussion

### 4.1. Characteristics of the Sample

This is the first study to ever study the presence of disordered eating attitudes among patients with celiac disease (CD) in Brazil. Regarding the final sample, it was noted that it mostly comprised women, similar to other studies conducted in Brazil [30,34].

Although genetic mechanisms are not well defined, it is noteworthy that CD occurs most preeminently in women, with an average ratio of 2.8:1 in relation with men [35]. A recent theory indicates that several factors may contribute to gender differences in autoimmune diseases, such as the exposure to environmental agents; endogenous hormones; differences in biology, such as pregnancy and menstruation; and epigenetic modifications related to chromosomes [36,37,38]. In addition, a study concluded that female patients with celiac disease carry alterations regarding haplotypes DQ2/DQ8, which are related to the occurrence of celiac disease, more frequently than men [37].

Another possibility is that men tend to obtain a CD diagnosis later than women, given that men also tend to seek less health services compared to women [39,40].

It is also important to highlight that in Brazil’s public healthcare, the diagnosis of CD is infrequent or completely unavailable; in this sense, one of the main reflections in the composition of the sample is the number of respondents with an average monthly income above BRL 5000.01 (USD 1038.71) [41,42]. In Brazil, the average household income is BRL 2800.00 (Around USD 582), so it is possible that only the population with higher monthly income has access to diagnostic tests for celiac disease, contributing to the increased share of this population in the included sample [43]. This effect was also noted regarding the place of living of the respondents. In Brazil, the higher gross domestic product (GDP) is concentrated among both South and Southeast regions, both regions with the highest amounts of respondents [43]. However, the increased access to higher education in this parcel of the population might have influenced the increased number of respondents with at least an undergraduate degree [43]. This fact also explains the lower percentage of responses in the North and Northeast regions of Brazil, regions whose GDP is the lowest in the country, possibly reflecting on diagnoses and lines of care in the context of CD.

However, the trend related to the distribution of body composition by income did not follow the pattern observed in the country as a whole. In general, the Brazilian population has a body composition closer to overweight and obese in strata with lower monthly income; on the other hand, more than half of the studied sample presented adequate weight (60%) [44,45].

A possible explanation for this result may be related to the eating attitude of celiac in Brazil. In general, results from research related to the overall quality of life of celiac demonstrate that Brazilian celiac have good results regarding the understanding of the disease and the quality of the diet necessary to control it [30,34,42].

### 4.2. Scores Obtained from the Self-Administered Online Questionnaire

In our sample, we reached only 13 responses from men, and none obtained a score greater than 21, demonstrating no disordered eating attitudes associated with an eating disorder [32]. There are few studies on disordered attitudes in men only, as there is a persistent view over time that eating disorders are linked to the female gender, which led to an underestimation of disordered eating attitudes and eating disorders in men [46,47]. It is notable in the studies regarding disordered eating attitudes that most of them have small clinical samples, the frequent use of diagnosis tools adapted for women, and especially the discomfort of male patients in resorting to services that are aimed mainly at women, as it is the case in Eds [48].

Gender differences reported within the literature depend on the specific symptoms of the eating disorder. Girls or women are more likely than boys or men to report weight dissatisfaction, dieting for weight control, and purge use, but they are just as likely or less likely than boys or men to report binge eating and use excessive exercise for weight control [49,50]. However, it has been shown that women are more likely than men to report body-checking attitudes, such as ritualistic weighing or trying on special clothing to check how it fits [47].

Regarding the included sample, when separating the participants by age, average monthly income, and educational level, it was noted that scores classified as disordered eating attitudes were not obtained; nevertheless, respondents with an educational level of high school or with an undergraduate degree presented higher scores in comparison to participants with at least a graduate-level degree.

In general, lower levels of education (below high school) are already associated with risk factors for the development of eating disorders, with the main theory being the fact that, associated with lower education, there is also evidence of lower monthly income and impaired access to health services [51,52].

However, some studies show that an undergraduate degree obtained from college courses negatively influence stress and mental health in general in groups of people with adequate weight and who are overweight and obese, thus increasing the risk of developing eating disorders [53,54]. In this sense, although the scores obtained are not sufficient to be considered associated with disordered eating attitudes, it is important to emphasize that the mentioned groups are at greater risk compared to the group of graduates in addition to the score being close to the point cutting stipulated by the instrument [27,32].

However, a critical point verified from the analyses is related to the BMI of the participants. In our research, overweight and obese participants presented scores classified as a disordered eating attitude (>21), with significantly higher scores in comparison to the remaining groups. Concern about weight is a common constant within the biopsychosocial process of developing eating disorders [55]; in addition, in our study, a considerable number of respondents referred to being constantly worried about gaining weight (Table 3). Frequently and more prominently in women, studies report social pressures related to the presented body composition, with individuals reporting impaired mental health, self-esteem, reduced job opportunities, social life, and difficulties in relationships [54,56].

In this sense, the occurrence of disordered eating attitudes increases since they appear as a “quick” alternative to obtain weight loss and satisfactory aesthetic results [57]. Although it is important to highlight that in addition to these attitudes being unhealthy, they very commonly produce effects contrary to the desired, contributing as an important risk factor in the increase in the prevalence of being overweight and obese [53,56,57].

As a fact, studies demonstrate that eating disorders, such as binge eating disorder (BED) and strict dieting, are common antecedents in obese women, thus supporting the results found among our sample [58,59,60,61]. Regarding items related to BED in the applied instrument, it was noted that the response frequency to item 25 (I like to try new high-calorie foods) was higher in the population with the highest score on the instrument, suggesting a critical point of risk.

In comparison with other studies conducted in Brazil that used the same instrument but in populations of women without celiac disease, the results showed frequencies of disordered eating attitudes varying between 16.5% and 30.1%, with no differences between different body mass indexes among respondents [52,62]. In this sense, our study presented a higher frequency for disordered eating attitudes, supporting our hypothesis that patients with celiac disease are more prone to develop this kind of issue given the nature of the restrictive treatment. However, more studies comparing populations with and without celiac disease are needed to better support this theory.

In celiac disease specifically, studies performed in other countries already show a higher prevalence of disordered eating attitudes compared to the general population, both among men and women [29].

A theory is that, with this condition, patients often a need to monitor the gluten content of foods along with fears about the effectiveness of the diet and concerns about preventing symptoms [63,64,65]. A point that corroborates this theory is the frequency of “Always” answers to question 3 (I feel worried about food) and the frequency of “Often” to question 19 (I demonstrate self-control in relation to food) of the applied questionnaire (Table 3), which relates with the constant concern with food, suggesting a critical attitude point within the Brazilian celiac community.

In addition, it is noticeable that untreated gastrointestinal symptoms can trigger an aversion to food, which can influence disordered attitudes and attitude [19]. The Satherley, Higgs, and Howard (2017) theoretical model suggests two pathways of eating disorders in gastrointestinal diseases, one of which is the post-diagnosis weight gain experiment [66]. This can also be demonstrated in the higher frequency of answers (always, often, and sometimes) to question 1 (I am terrified with the idea of getting fat) and 14 (I am worried about the idea of having fat in my body) of the applied questionnaire (Table 3).

People affected with gastrointestinal diseases, such as the celiac disease, may believe that their diet causes them to gain weight, which leads to dysfunctional diet beliefs and attitudes, such as a lack of adherence to the dietary regimen, ongoing gastrointestinal symptoms, and psychological distress [67]. In the literature, there is a study that describes three cases in which the concern with weight increased after starting a gluten-free diet [68].

Indeed, such a theory is supported by the nutritional quality of commercial gluten-free foods. In general, it is noted that gluten-free foods often use high-glycemic index flours, with a high carbohydrate content and less dietary fiber compared to their gluten-containing counterparts [69,70,71]. In addition, a higher energy content is evident given that, often, a higher quantity of fat is implemented as an ingredient to improve sensory and technological characteristics of gluten-free products [72].

In this sense, the adoption of public policies stands out, both with regard to access to healthier gluten-free products and the creation of policies that seek to prevent and treat disordered eating attitudes associated with eating disorders in people with celiac disease in Brazil.

Regarding the limitations of this study, it is important to highlight that the instrument did not assess whether the participants were following a strictly gluten-free diet. Furthermore, the presence of other comorbidities that require attention to nutrition, such as Type 1 diabetes mellitus, was not assessed in this study and may therefore have influenced a small portion of respondents given the concomitant occurrence of this disease with CD. In addition, although disordered eating attitudes are associated with a greater risk of developing eating disorders, our study did not utilize instruments for the diagnosis of any eating disorder. 

## 5. Conclusions

In conclusion, we believe that our study brings together an important research area for the first time in Brazil. A total of 36.1% of the included sample presented scores classified as a disordered eating attitude; however, the stratification of the data showed that women with celiac disease and BMIs classified as overweight and obese presented higher scores in comparison to other sociodemographic characteristics. It is important to note that disordered eating attitudes are associated with a higher risk of developing eating disorders; thus, our study highlights the need for the implementation of public health policies directed to this public in order to prevent and treat those disordered eating attitudes.

## Figures and Tables

**Table 1:** Sociodemographic and BMI data of the study participants.

Variable	Number	% of the Sample
Gender (F or M)		
Female	371	96.36%
Male	13	3.37%
Average Monthly Income (BRL and USD)		
Up to 3000.00 BRL (USD 623.00)	76	19.94%
3000.01 to 5000 BRL (USD 623.23 to 1038.71)	86	22.33%
5000.01 to 10,000.01 BRL (USD 1038.71 to 2077.43)	112	29.09%
>10,000.01 BRL (USD 2077.43)	111	28.83%
Age (Years)		
18 to 24 years old	110	28.57%
25 to 34 years old	136	35.32%
35 to 44 years old	76	19.74%
45 to 54 years old	43	11.16%
>55 years old	20	5.19%
Educational Level		
Up to High School	55	14.28%
Undergraduate Level (B.Sc)	189	49.09%
Graduate Level or Above (Post-Graduation, Masters degree, or Ph.D.)	141	29.61%
Average BMI (Kg/m^2^)		
Underweight (<18.5 Kg/m^2^)	18.7 ± 11.29 Kg/m^2^; *n* = 30	7.79%
Normal Weight (≥18.5 Kg/m^2^ < 25 Kg/m^2^)	17.23 ± 9.13 Kg/m^2^; *n* = 231	60%
Overweight (≥25 Kg/m^2^ < 30 Kg/m^2^)	21.44 ± 10.09 Kg/m^2^; *n* = 84	21.81%
Obesity (≥30 Kg/m^2^)	24.85 ± 11.28 Kg/m^2^; *n* = 40	10.38%

**Table 2:** Average score collected from the self-administered online EAT-26 questionnaire.

Variable	Average Score	p Value
Gender (F or M)		
Female	19.25 ± 10.05 ^b^	<0.001 *
Male	11.85 ± 3.59 ^a^	
Average Monthly Income (BRL and USD)		
Up to 3000.00 BRL (USD 623.00)	20.66 ± 11.61 ^a^	0.355 **
3000.01 to 5000 BRL (USD 623.23 to 1038.71)	17.84 ± 8.57 ^a^	
5000.01 to 10,000.01 BRL (USD 1038.71 to 2077.43)	18.83 ± 9.39 ^a^	
>10,000.01 BRL (USD 2077.43)	19.14 ± 10.61 ^a^	
Age (Years)		
18 to 24 years old	19.19 ± 10.51 ^a^	0.183 **
25 to 34 years old	19.82 ± 10.82 ^a^	
35 to 44 years old	16.70 ± 8.60 ^a^	
45 to 54 years old	20.72 ± 9.78 ^a^	
>55 years old	18.55 ± 6.47 ^a^	
Educational Level		
Up to High School	20.02 ± 10.27 ^b^	0.025 **
Undergraduate Level (B.Sc)	20.14 ± 10.47 ^b^	
Graduate Level or Above (Post-Graduation, Masters degree, or Ph.D.)	17.23 ± 9.18 ^a^	
Average Score by BMI (Kg/m^2^)		
Underweight (<18.5 Kg/m^2^)	18.70 ± 11.23 ^a^	<0.001 **
Adequate Weight (≥18.5 Kg/m^2^ < 25 Kg/m^2^)	17.24 ± 9.13 ^a^	
Overweight (≥25 Kg/m^2^ < 30 Kg/m^2^)	21.44 ±10.10 ^ab^	
Obesity (≥30 Kg/m^2^)	24.85 ± 11.28 ^b^	

* Non-paired Student’s *t*-test; ** ANOVA with Tukey’s post hoc test. Values in the same category with different superscript letters presented significant differences (*p* < 0.05).

**Table 3:** Absolute frequencies of answers per item in the self-administered questionnaire of the groups with normal and disordered eating attitudes.

Item	Always	Usually	Often	Sometimes	Rarely	Never	Always	Usually	Often	Sometimes	Rarely	Never
	Overall Score < 21; Frequency in (%)						Overall Score > 21; Frequency in (%)					
1	10.6	16.3	25.6	12.2	13.8	21.5	25.5	51.8	31.7	9.4	2.2	1.4
2	0.0	1.2	8.1	14.6	28.5	47.6	0.5	1.4	13.7	29.5	9.4	23.0
3	32.5	39.0	15.9	6.1	3.7	2.8	44.4	65.5	23.0	8.6	0.7	1.4
4	1.2	6.1	13.8	19.9	19.1	39.8	10.1	25.9	21.6	18.7	12.2	8.6
5	10.2	17.9	21.1	15.4	16.3	19.1	14.0	20.9	18.0	23.0	16.5	8.6
6	1.6	8.1	14.2	13.0	17.1	45.9	6.2	14.4	24.5	27.3	7.9	12.9
7	2.0	3.3	12.2	14.2	26.4	41.9	2.6	3.6	17.3	33.8	15.8	12.9
8	4.5	7.7	13.0	11.0	18.7	45.1	7.0	11.5	12.2	17.3	12.2	13.7
9	0.0	0.0	0.0	1.2	5.3	93.5	0.0	0.0	2.2	5.0	2.9	9.4
10	0.4	1.6	11.8	13.0	23.2	50.0	6.2	16.5	20.9	25.2	11.5	15.1
11	4.5	8.1	21.1	13.0	16.7	36.6	20.5	48.9	18.7	16.5	2.9	5.0
12	3.7	7.7	18.7	8.5	19.1	42.3	17.4	41.7	17.3	21.6	6.5	5.0
13	5.7	10.2	14.6	15.4	16.7	37.4	6.8	8.6	5.8	15.1	9.4	12.2
14	5.7	11.0	22.4	12.6	23.2	25.2	18.7	41.7	30.2	18.0	2.9	3.6
15	9.3	9.3	19.9	15.0	24.4	22.0	11.7	15.8	17.3	18.7	14.4	15.8
16	2.0	12.6	17.5	18.3	24.4	25.2	4.2	7.9	25.9	24.5	12.9	16.5
17	0.8	3.3	12.2	11.4	26.4	45.9	1.3	2.2	10.8	25.2	10.1	22.3
18	5.3	13.8	17.5	13.8	13.0	36.6	13.2	27.3	25.2	18.7	12.9	7.2
19	22.8	41.5	20.7	8.5	4.5	2.0	19.0	12.2	29.5	28.8	12.2	13.7
20	2.4	3.3	14.6	13.8	24.0	41.9	4.7	8.6	12.9	20.1	12.2	14.4
21	2.4	13.8	21.1	21.5	26.4	14.6	8.1	18.0	30.2	25.9	12.9	7.9
22	0.8	4.1	21.1	15.0	18.3	40.7	4.4	10.8	23.7	28.8	11.5	11.5
23	0.0	2.4	8.5	11.4	19.9	57.7	5.7	15.8	20.9	22.3	11.5	15.8
24	0.0	0.4	6.9	7.7	13.8	71.1	0.8	2.2	15.8	13.7	10.8	17.3
25	3.7	9.8	30.1	21.5	18.3	16.7	5.5	8.6	14.4	27.3	18.0	23.0
26	0.0	1.2	1.6	3.7	7.3	86.2	1.3	3.6	3.6	10.1	5.8	15.1

## Data Availability

Data are contained within the article and supplementary materials.

## Supplementary Materials

The following supporting information can be downloaded at: https://www.mdpi.com/article/10.3390/nu15224796/s1, Table S1: Full data collected from the self-administered translated to Brazilian Portuguese version of the EAT-26 questionnaire.

## Appendix A

Validated Version of the EAT-26 questionnaire [27].

Name:___________________________________________________________

Age:______ Weight:______ Height:_____

**Please Answer All the Following Items**	**Always**	**Usually**	**Often**	**Sometimes**	**Rarely**	**Never**
1—I’m terrified of the idea of gaining weight						
2—I avoid eating when I’m hungry						
3—I feel worried about food						
4—Continuing to overeat makes me feel like I can’t stop						
5—I cut my food into small pieces						
6—I pay attention to the number of calories in the food I eat						
7—I particularly avoid foods rich in carbohydrates (e.g., bread, rice, potatoes, etc.)						
8—I feel like others would like me to eat more.						
9—Vomiting after eating						
10—I feel extremely guilty after eating						
11—I worry about wanting to be thinner						
12—I think about burning extra calories when I exercise						
13—People think I’m too thin						
14—I worry about having fat on my body						
15—It takes me longer to eat my meals than other people						
16—I avoid eating foods that contain sugar						
17—I usually eat diet foods						
18—I feel like food controls my life						
19—I demonstrate self-control around food						
20—I feel like others pressure me to eat						
21—I spend a lot of time thinking about eating						
22—I feel discomfort after eating sweets						
23—I follow weight loss regimes						
24—I like feeling my stomach empty						
25—I like to try new high-calorie foods						
26—I feel like vomiting after meals						
