# Supplementary material for: Eating Attitudes of Patients with Celiac Disease in Brazil: A Nationwide Assessment with the EAT-26 Instrument
Source: Nutrients. 2023 Nov 16;15(22):4796. doi: 10.3390/nu15224796 (PMC10674570; doi:10.3390/nu15224796)
Supplement: Supplementary file 1 [file nutrients-15-04796-s001.zip › nutrients-2702337-supplementary.pdf]

Table S1. Full description of answers collected from the online self-administered version of the Brazilian version of the EAT-26 Questionnaire.

Number	1.I am terrified about being overweight	2. I Avoid eating when I am Hungry	3. I Find Myself preoccupied with food	4. I Have gone on eating binges where I feel that I may not be able to stop	5. I Cut my food into small pieces	6. I'm aware of the calorie content of foods that I eat	7. I particulary avoid food with a high carbohydrate content (i.e bread, rice, potatoes etc.)	8. I feel that other would prefer if I ate more	9. I vomit after I have eaten	10. I feel extremely guilty after eating	11. I am preoccupied with a desire to be thinner	12. I think about burning up calories when I exercise	13. Other people think that I am too thin	14. I am preoccupied with the thought of having fat on my body	15. I take longer than others to eat my meals	16. I avoid foods with sugar in them	17. I eat diet foods	18. I feel that food controls my life	19. I display self-control around food	20. I feel that others pressure me to eat	21. I give too much time and thought to food	22. I feel uncomfortable after eating sweets	23. I engage in dieting behavior	25. I like my stomach to be empty	25. I enjoy trying new rich foods	26. I have to impulse to vomit after meals	Score
1	Always	Usually	Always	Usually	Always	Always	Usually	Usually	Sometimes	Usually	Always	Always	Usually	Always	Usually	Usually	Usually	Always	Usually	Usually	Always	Always	Usually	Always	Rarely	Usually	60
2	Always	Usually	Usually	Always	Usually	Usually	Usually	Usually	Sometimes	Always	Always	Always	Sometimes	Always	Sometimes	Usually	Usually	Always	Always	Always	Always	Usually	Usually	Usually	Usually	Usually	59
3	Usually	Sometimes	Always	Always	Always	Always	Always	Sometimes	Sometimes	Sometimes	Always	Always	Sometimes	Always	Sometimes	Always	Usually	Usually	Usually	Sometimes	Always	Usually	Always	Usually	Rarely	Sometimes	53
4	Always	Usually	Always	Always	Sometimes	Always	Usually	Never	Sometimes	Always	Always	Always	Never	Always	Never	Usually	Sometimes	Always	Never	Sometimes	Always	Always	Always	Usually	Sometimes	Always	52
5	Always	Sometimes	Always	Sometimes	Usually	Always	Usually	Always	Never	Usually	Always	Always	Always	Always	Always	Usually	Sometimes	Always	Sometimes	Usually	Usually	Usually	Usually	Usually	Never	Never	52
6	Sometimes	Sometimes	Always	Always	Sometimes	Always	Sometimes	Always	Sometimes	Usually	Always	Always	Always	Usually	Always	Always	Never	Always	Sometimes	Always	Always	Usually	Sometimes	Sometimes	Never	Always	53
7	Always	Usually	Always	Always	Sometimes	Sometimes	Always	Sometimes	Rarely	Always	Always	Sometimes	Never	Always	Always	Always	Always	Always	Usually	Sometimes	Always	Always	Always	Sometimes	Sometimes	Sometimes	54
8	Always	Sometimes	Always	Always	Rarely	Always	Sometimes	Never	Rarely	Usually	Always	Always	Never	Always	Sometimes	Sometimes	Always	Always	Usually	Sometimes	Always	Usually	Usually	Usually	Sometimes	Sometimes	47
9	Always	Usually	Usually	Always	Always	Sometimes	Sometimes	Never	Usually	Usually	Always	Always	Never	Always	Sometimes	Sometimes	Sometimes	Usually	Sometimes	Sometimes	Sometimes	Usually	Always	Usually	Sometimes	Always	47
10	Always	Always	Always	Always	Sometimes	Usually	Always	Sometimes	Rarely	Always	Always	Always	Never	Always	Sometimes	Sometimes	Sometimes	Sometimes	Sometimes	Sometimes	Usually	Always	Always	Always	Sometimes	Sometimes	50
11	Sometimes	Sometimes	Always	Rarely	Sometimes	Usually	Sometimes	Always	Never	Usually	Always	Always	Always	Always	Sometimes	Usually	Usually	Usually	Always	Sometimes	Usually	Usually	Usually	Usually	Sometimes	Never	46
12	Always	Sometimes	Always	Always	Rarely	Usually	Usually	Never	Never	Always	Always	Always	Never	Always	Sometimes	Sometimes	Always	Always	Rarely	Never	Usually	Always	Always	Never	Always	Never	45
13	Always	Usually	Always	Always	Usually	Sometimes	Sometimes	Sometimes	Never	Usually	Always	Always	Rarely	Always	Rarely	Sometimes	Sometimes	Usually	Sometimes	Sometimes	Always	Usually	Usually	Rarely	Always	Rarely	43
14	Always	Sometimes	Always	Sometimes	Always	Sometimes	Always	Always	Never	Sometimes	Always	Sometimes	Sometimes	Always	Always	Sometimes	Never	Always	Sometimes	Always	Sometimes	Sometimes	Always	Rarely	Never	Never	43
15	Usually	Sometimes	Always	Sometimes	Always	Sometimes	Sometimes	Sometimes	Rarely	Sometimes	Usually	Usually	Never	Always	Sometimes	Usually	Never	Usually	Usually	Always	Always	Sometimes	Always	Usually	Rarely	Rarely	40
16	Usually	Sometimes	Always	Always	Sometimes	Never	Sometimes	Never	Usually	Always	Always	Always	Sometimes	Always	Rarely	Sometimes	Sometimes	Sometimes	Never	Never	Sometimes	Usually	Usually	Sometimes	Never	Always	38
17	Always	Rarely	Always	Sometimes	Usually	Usually	Rarely	Sometimes	Never	Sometimes	Always	Always	Sometimes	Always	Always	Never	Sometimes	Usually	Sometimes	Rarely	Usually	Never	Always	Usually	Usually	Rarely	39
18	Always	Sometimes	Always	Always	Usually	Usually	Sometimes	Never	Rarely	Always	Always	Always	Never	Always	Never	Rarely	Sometimes	Usually	Rarely	Never	Always	Sometimes	Always	Sometimes	Usually	Rarely	40

19	Never	Usually	Always	Never	Always	Always	Never	Always	Never	Never	Never	Never	Always	Never	Always	Rarely	Sometimes	Always	Sometimes	Always	Always	Usually	Never	Usually	Usually	Sometimes	38
20	Usually	Sometimes	Sometimes	Always	Always	Sometimes	Sometimes	Always	Never	Always	Always	Always	Never	Always	Sometimes	Rarely	Sometimes	Sometimes	Never	Sometimes	Sometimes	Sometimes	Usually	Usually	Sometimes	Never	38
21	Always	Sometimes	Always	Always	Always	Never	Sometimes	Never	Sometimes	Always	Always	Always	Never	Usually	Never	Sometimes	Usually	Always	Sometimes	Rarely	Usually	Usually	Always	Usually	Sometimes	Sometimes	44
22	Usually	Sometimes	Usually	Sometimes	Sometimes	Always	Sometimes	Usually	Never	Sometimes	Sometimes	Usually	Always	Always	Usually	Usually	Usually	Always	Usually	Always	Sometimes	Usually	Never	Never	Sometimes	Never	41
23	Always	Usually	Always	Always	Never	Sometimes	Sometimes	Never	Never	Always	Always	Usually	Never	Always	Always	Sometimes	Sometimes	Always	Sometimes	Never	Sometimes	Sometimes	Sometimes	Rarely	Sometimes	Never	37
24	Always	Sometimes	Always	Usually	Sometimes	Always	Usually	Never	Never	Usually	Always	Always	Never	Always	Rarely	Usually	Sometimes	Usually	Usually	Sometimes	Sometimes	Usually	Sometimes	Never	Never	Never	38
25	Always	Usually	Sometimes	Usually	Sometimes	Sometimes	Usually	Sometimes	Never	Always	Always	Usually	Never	Always	Sometimes	Sometimes	Rarely	Sometimes	Sometimes	Rarely	Usually	Sometimes	Always	Usually	Always	Never	39
26	Always	Sometimes	Usually	Usually	Never	Usually	Never	Sometimes	Rarely	Usually	Always	Always	Never	Always	Rarely	Usually	Usually	Sometimes	Sometimes	Rarely	Usually	Usually	Usually	Sometimes	Sometimes	Sometimes	37
27	Usually	Never	Always	Usually	Usually	Always	Never	Usually	Never	Sometimes	Never	Always	Sometimes	Always	Never	Always	Never	Always	Sometimes	Usually	Usually	Usually	Never	Never	Usually	Never	37
28	Always	Sometimes	Always	Always	Sometimes	Usually	Sometimes	Sometimes	Never	Always	Usually	Always	Rarely	Sometimes	Sometimes	Sometimes	Sometimes	Usually	Rarely	Sometimes	Always	Usually	Always	Sometimes	Rarely	Never	39
29	Usually	Sometimes	Sometimes	Usually	Never	Usually	Sometimes	Always	Sometimes	Usually	Usually	Always	Always	Always	Sometimes	Never	Never	Sometimes	Always	Always	Rarely	Sometimes	Sometimes	Never	Usually	Usually	40
30	Usually	Sometimes	Always	Always	Sometimes	Never	Sometimes	Sometimes	Sometimes	Always	Always	Usually	Rarely	Always	Rarely	Rarely	Rarely	Always	Rarely	Usually	Always	Usually	Always	Sometimes	Usually	Sometimes	41
31	Usually	Rarely	Always	Sometimes	Sometimes	Sometimes	Usually	Sometimes	Never	Sometimes	Always	Always	Rarely	Always	Usually	Usually	Sometimes	Sometimes	Usually	Rarely	Usually	Sometimes	Usually	Sometimes	Always	Never	38
32	Always	Sometimes	Always	Rarely	Sometimes	Usually	Sometimes	Always	Never	Rarely	Usually	Always	Usually	Usually	Sometimes	Sometimes	Sometimes	Always	Usually	Usually	Usually	Sometimes	Sometimes	Rarely	Rarely	Never	37
33	Always	Sometimes	Usually	Usually	Usually	Sometimes	Usually	Usually	Never	Usually	Always	Always	Usually	Always	Sometimes	Sometimes	Never	Sometimes	Usually	Sometimes	Sometimes	Sometimes	Usually	Rarely	Rarely	Rarely	38
34	Always	Sometimes	Always	Always	Sometimes	Sometimes	Rarely	Usually	Never	Always	Always	Always	Never	Always	Rarely	Sometimes	Sometimes	Sometimes	Sometimes	Always	Sometimes	Sometimes	Always	Sometimes	Sometimes	Sometimes	41
35	Usually	Sometimes	Always	Usually	Rarely	Rarely	Sometimes	Usually	Rarely	Usually	Sometimes	Always	Usually	Usually	Always	Sometimes	Never	Always	Sometimes	Usually	Always	Sometimes	Sometimes	Never	Sometimes	Rarely	37
36	Always	Never	Always	Always	Always	Never	Never	Never	Never	Always	Always	Never	Never	Always	Sometimes	Never	Never	Always	Never	Never	Always	Rarely	Always	Never	Always	Never	34
37	Usually	Rarely	Always	Sometimes	Rarely	Usually	Usually	Never	Never	Usually	Always	Always	Never	Always	Never	Sometimes	Sometimes	Sometimes	Sometimes	Sometimes	Always	Usually	Sometimes	Usually	Sometimes	Never	35
38	Always	Sometimes	Always	Always	Never	Sometimes	Never	Sometimes	Never	Sometimes	Always	Always	Never	Always	Never	Never	Never	Always	Sometimes	Never	Always	Never	Sometimes	Never	Always	Sometimes	34
39	Usually	Never	Always	Always	Always	Always	Sometimes	Usually	Never	Never	Never	Always	Always	Never	Rarely	Always	Never	Sometimes	Always	Sometimes	Usually	Rarely	Never	Sometimes	Never	Never	34
40	Always	Never	Always	Sometimes	Rarely	Rarely	Sometimes	Rarely	Never	Sometimes	Sometimes	Sometimes	Sometimes	Usually	Usually	Usually	Usually	Usually	Usually	Usually	Usually	Usually	Usually	Rarely	Sometimes	Sometimes	34
41	Always	Usually	Always	Always	Always	Sometimes	Sometimes	Never	Never	Sometimes	Sometimes	Sometimes	Never	Always	Never	Usually	Never	Always	Sometimes	Never	Sometimes	Never	Always	Never	Sometimes	Never	33
42	Always	Rarely	Usually	Always	Never	Sometimes	Never	Never	Usually	Always	Always	Sometimes	Never	Usually	Never	Never	Never	Always	Never	Never	Always	Always	Rarely	Never	Sometimes	Always	33

43	Sometimes	Never	Always	Rarely	Sometimes	Sometimes	Never	Always	Sometimes	Rarely	Sometimes	Sometimes	Sometimes	Sometimes	Sometimes	Always	Sometimes	Usually	Always	Always	Sometimes	Rarely	Sometimes	Never	Always	Sometimes	33
44	Usually	Usually	Usually	Sometimes	Always	Sometimes	Sometimes	Never	Rarely	Usually	Always	Always	Sometimes	Usually	Sometimes	Sometimes	Never	Never	Sometimes	Never	Sometimes	Always	Usually	Sometimes	Never	Usually	35
45	Usually	Usually	Usually	Sometimes	Usually	Sometimes	Sometimes	Always	Sometimes	Always	Sometimes	Always	Sometimes	Sometimes	Always	Sometimes	Sometimes	Usually	Sometimes	Sometimes	Sometimes	Usually	Sometimes	Rarely	Sometimes	Sometimes	39
46	Always	Sometimes	Always	Always	Always	Sometimes	Sometimes	Rarely	Never	Always	Always	Always	Never	Always	Always	Sometimes	Rarely	Usually	Sometimes	Sometimes	Sometimes	Sometimes	Rarely	Never	Sometimes	Never	38
47	Rarely	Usually	Always	Never	Always	Usually	Sometimes	Sometimes	Never	Never	Never	Sometimes	Usually	Always	Always	Usually	Never	Never	Always	Sometimes	Rarely	Usually	Never	Always	Rarely	Never	32
48	Always	Sometimes	Sometimes	Usually	Usually	Usually	Sometimes	Rarely	Never	Usually	Always	Always	Never	Always	Usually	Rarely	Rarely	Sometimes	Rarely	Never	Usually	Sometimes	Sometimes	Sometimes	Sometimes	Sometimes	33
49	Always	Never	Always	Never	Always	Always	Usually	Sometimes	Never	Rarely	Sometimes	Always	Rarely	Sometimes	Usually	Always	Never	Rarely	Always	Sometimes	Sometimes	Usually	Sometimes	Rarely	Rarely	Never	33
50	Always	Usually	Always	Rarely	Never	Sometimes	Sometimes	Sometimes	Never	Sometimes	Usually	Usually	Rarely	Always	Sometimes	Usually	Rarely	Always	Usually	Sometimes	Rarely	Always	Sometimes	Rarely	Sometimes	Never	33
51	Usually	Sometimes	Sometimes	Always	Sometimes	Sometimes	Sometimes	Never	Rarely	Sometimes	Sometimes	Always	Never	Always	Sometimes	Usually	Rarely	Sometimes	Sometimes	Sometimes	Usually	Usually	Always	Usually	Sometimes	Rarely	34
52	Always	Rarely	Always	Never	Always	Usually	Sometimes	Never	Never	Rarely	Always	Sometimes	Never	Always	Usually	Sometimes	Never	Usually	Usually	Never	Never	Sometimes	Usually	Usually	Rarely	Never	31
53	Always	Never	Usually	Usually	Sometimes	Sometimes	Sometimes	Never	Never	Usually	Always	Usually	Never	Usually	Never	Never	Sometimes	Sometimes	Sometimes	Sometimes	Always	Sometimes	Usually	Usually	Sometimes	Rarely	32
54	Usually	Never	Always	Sometimes	Always	Usually	Sometimes	Never	Never	Usually	Sometimes	Always	Never	Always	Usually	Usually	Sometimes	Usually	Sometimes	Rarely	Sometimes	Sometimes	Sometimes	Never	Rarely	Rarely	32
55	Sometimes	Sometimes	Usually	Rarely	Rarely	Rarely	Rarely	Always	Sometimes	Sometimes	Always	Sometimes	Always	Always	Usually	Rarely	Rarely	Usually	Sometimes	Usually	Rarely	Sometimes	Sometimes	Usually	Rarely	Sometimes	31
56	Always	Never	Always	Never	Never	Always	Always	Sometimes	Never	Rarely	Always	Always	Sometimes	Always	Never	Sometimes	Sometimes	Never	Usually	Sometimes	Sometimes	Sometimes	Usually	Rarely	Sometimes	Never	33
57	Always	Never	Always	Usually	Always	Sometimes	Rarely	Rarely	Never	Usually	Always	Rarely	Never	Usually	Rarely	Sometimes	Usually	Always	Sometimes	Rarely	Usually	Usually	Sometimes	Never	Rarely	Never	31
58	Always	Rarely	Usually	Always	Sometimes	Usually	Never	Never	Never	Usually	Always	Sometimes	Never	Always	Rarely	Rarely	Never	Always	Rarely	Never	Always	Always	Sometimes	Never	Rarely	Never	30
59	Always	Sometimes	Always	Always	Sometimes	Sometimes	Sometimes	Never	Never	Always	Always	Sometimes	Never	Usually	Rarely	Rarely	Never	Usually	Rarely	Never	Usually	Rarely	Always	Rarely	Usually	Rarely	31
60	Always	Usually	Always	Sometimes	Rarely	Sometimes	Never	Sometimes	Never	Sometimes	Always	Sometimes	Rarely	Always	Sometimes	Sometimes	Rarely	Usually	Usually	Usually	Usually	Sometimes	Sometimes	Never	Rarely	Never	31
61	Always	Sometimes	Always	Sometimes	Sometimes	Always	Rarely	Rarely	Never	Usually	Always	Usually	Sometimes	Always	Sometimes	Never	Never	Usually	Sometimes	Never	Sometimes	Sometimes	Sometimes	Never	Rarely	Never	30
62	Always	Never	Always	Never	Never	Always	Never	Never	Never	Never	Always	Always	Never	Always	Never	Sometimes	Never	Always	Usually	Never	Never	Sometimes	Always	Never	Sometimes	Never	29
63	Always	Sometimes	Usually	Sometimes	Rarely	Sometimes	Sometimes	Sometimes	Never	Sometimes	Always	Always	Sometimes	Always	Always	Rarely	Never	Sometimes	Sometimes	Sometimes	Sometimes	Sometimes	Usually	Sometimes	Usually	Rarely	34
64	Always	Usually	Always	Usually	Usually	Sometimes	Rarely	Usually	Never	Usually	Usually	Sometimes	Rarely	Usually	Sometimes	Usually	Never	Sometimes	Rarely	Always	Sometimes	Sometimes	Sometimes	Sometimes	Rarely	Never	33
65	Sometimes	Never	Usually	Never	Never	Always	Sometimes	Sometimes	Never	Sometimes	Always	Always	Never	Always	Never	Usually	Never	Never	Always	Never	Never	Always	Usually	Sometimes	Rarely	Rarely	29
66	Always	Sometimes	Always	Usually	Usually	Sometimes	Sometimes	Usually	Never	Rarely	Sometimes	Rarely	Sometimes	Usually	Always	Sometimes	Sometimes	Sometimes	Usually	Sometimes	Sometimes	Usually	Sometimes	Usually	Sometimes	Never	35
67	Always	Sometimes	Sometimes	Never	Always	Never	Never	Sometimes	Never	Sometimes	Rarely	Never	Always	Always	Usually	Usually	Sometimes	Usually	Sometimes	Sometimes	Usually	Always	Rarely	Usually	Never	Rarely	32
68	Always	Never	Never	Always	Usually	Rarely	Never	Never	Never	Always	Always	Never	Never	Sometimes	Rarely	Rarely	Never	Always	Rarely	Never	Always	Always	Usually	Never	Usually	Never	28

69	Usually	Sometimes	Always	Usually	Sometimes	Rarely	Sometimes	Rarely	Never	Usually	Never	Always	Never	Always	Usually	Usually	Never	Sometimes	Sometimes	Rarely	Sometimes	Sometimes	Usually	Sometimes	Sometimes	Never	30
70	Always	Sometimes	Always	Never	Always	Usually	Sometimes	Never	Never	Sometimes	Always	Never	Sometimes	Sometimes	Usually	Usually	Usually	Rarely	Usually	Never	Sometimes	Rarely	Sometimes	Rarely	Rarely	Never	29
71	Always	Usually	Usually	Sometimes	Sometimes	Sometimes	Sometimes	Rarely	Rarely	Sometimes	Usually	Usually	Never	Usually	Sometimes	Usually	Sometimes	Sometimes	Sometimes	Sometimes	Sometimes	Usually	Sometimes	Sometimes	Sometimes	Never	31
72	Always	Never	Always	Always	Never	Never	Rarely	Sometimes	Never	Always	Always	Always	Never	Always	Sometimes	Rarely	Never	Always	Rarely	Never	Usually	Sometimes	Rarely	Never	Sometimes	Never	30
73	Usually	Rarely	Usually	Sometimes	Sometimes	Sometimes	Sometimes	Rarely	Never	Sometimes	Usually	Sometimes	Sometimes	Usually	Usually	Always	Sometimes	Rarely	Usually	Sometimes	Sometimes	Always	Sometimes	Sometimes	Rarely	Sometimes	31
74	Always	Sometimes	Always	Always	Never	Never	Sometimes	Rarely	Never	Sometimes	Always	Sometimes	Never	Always	Sometimes	Never	Sometimes	Never	Sometimes	Sometimes	Usually	Sometimes	Sometimes	Sometimes	Usually	Sometimes	31
75	Usually	Rarely	Usually	Usually	Rarely	Sometimes	Sometimes	Sometimes	Never	Sometimes	Usually	Always	Sometimes	Usually	Always	Rarely	Sometimes	Sometimes	Sometimes	Sometimes	Usually	Rarely	Usually	Never	Sometimes	Never	30
76	Sometimes	Sometimes	Always	Sometimes	Usually	Sometimes	Usually	Usually	Rarely	Rarely	Sometimes	Sometimes	Never	Sometimes	Rarely	Usually	Sometimes	Sometimes	Sometimes	Usually	Sometimes	Usually	Sometimes	Usually	Sometimes	Never	30
77	Always	Never	Usually	Usually	Usually	Usually	Sometimes	Never	Never	Rarely	Always	Sometimes	Never	Sometimes	Usually	Never	Sometimes	Never	Usually	Usually	Sometimes	Rarely	Never	Never	Usually	Never	27
78	Always	Never	Always	Sometimes	Usually	Sometimes	Sometimes	Never	Never	Rarely	Always	Usually	Never	Usually	Never	Sometimes	Sometimes	Sometimes	Usually	Never	Usually	Rarely	Always	Never	Sometimes	Never	29
79	Always	Sometimes	Always	Sometimes	Usually	Usually	Usually	Sometimes	Never	Rarely	Sometimes	Usually	Rarely	Usually	Usually	Sometimes	Rarely	Rarely	Usually	Rarely	Rarely	Sometimes	Usually	Rarely	Rarely	Never	28
80	Always	Sometimes	Always	Sometimes	Always	Never	Sometimes	Never	Never	Never	Sometimes	Always	Never	Sometimes	Never	Sometimes	Never	Never	Always	Never	Never	Sometimes	Usually	Never	Sometimes	Never	25
81	Usually	Sometimes	Always	Sometimes	Usually	Rarely	Sometimes	Rarely	Never	Usually	Always	Sometimes	Never	Sometimes	Always	Sometimes	Never	Usually	Sometimes	Sometimes	Always	Sometimes	Sometimes	Sometimes	Sometimes	Sometimes	33
82	Always	Rarely	Sometimes	Usually	Sometimes	Sometimes	Sometimes	Usually	Never	Sometimes	Always	Usually	Never	Sometimes	Sometimes	Sometimes	Rarely	Always	Rarely	Sometimes	Usually	Sometimes	Sometimes	Sometimes	Rarely	Rarely	29
83	Always	Sometimes	Sometimes	Never	Sometimes	Sometimes	Sometimes	Sometimes	Never	Sometimes	Sometimes	Usually	Sometimes	Always	Sometimes	Sometimes	Sometimes	Sometimes	Always	Sometimes	Sometimes	Sometimes	Sometimes	Never	Sometimes	Never	29
84	Usually	Never	Always	Sometimes	Sometimes	Usually	Usually	Always	Never	Never	Sometimes	Sometimes	Rarely	Always	Usually	Usually	Never	Never	Usually	Rarely	Sometimes	Rarely	Rarely	Never	Rarely	Never	26
85	Usually	Never	Usually	Usually	Sometimes	Sometimes	Usually	Never	Never	Sometimes	Usually	Sometimes	Never	Sometimes	Never	Usually	Sometimes	Sometimes	Sometimes	Sometimes	Sometimes	Usually	Usually	Never	Sometimes	Never	27
86	Never	Never	Always	Never	Always	Never	Never	Always	Never	Never	Never	Never	Always	Never	Never	Never	Never	Always	Always	Always	Sometimes	Never	Never	Never	Usually	Sometimes	25
87	Sometimes	Never	Always	Sometimes	Sometimes	Usually	Never	Sometimes	Never	Never	Never	Sometimes	Rarely	Sometimes	Always	Always	Usually	Always	Always	Never	Usually	Sometimes	Never	Never	Sometimes	Never	29
88	Usually	Sometimes	Usually	Sometimes	Sometimes	Sometimes	Sometimes	Never	Never	Sometimes	Usually	Always	Rarely	Usually	Usually	Sometimes	Rarely	Usually	Sometimes	Never	Usually	Never	Rarely	Never	Sometimes	Never	26
89	Usually	Sometimes	Usually	Sometimes	Sometimes	Always	Sometimes	Rarely	Never	Sometimes	Usually	Usually	Rarely	Usually	Sometimes	Usually	Sometimes	Sometimes	Sometimes	Rarely	Sometimes	Sometimes	Usually	Sometimes	Sometimes	Rarely	30
90	Usually	Rarely	Sometimes	Always	Usually	Sometimes	Sometimes	Sometimes	Never	Sometimes	Usually	Never	Never	Sometimes	Always	Rarely	Rarely	Always	Rarely	Never	Sometimes	Never	Sometimes	Sometimes	Always	Never	27
91	Sometimes	Always	Always	Never	Always	Sometimes	Sometimes	Sometimes	Never	Sometimes	Rarely	Sometimes	Sometimes	Rarely	Usually	Sometimes	Never	Never	Always	Sometimes	Never	Never	Never	Sometimes	Rarely	Never	24
92	Always	Never	Always	Always	Rarely	Rarely	Rarely	Never	Never	Always	Always	Always	Never	Usually	Sometimes	Rarely	Never	Sometimes	Sometimes	Rarely	Sometimes	Sometimes	Sometimes	Never	Sometimes	Never	27

93	Usually	Rarely	Always	Rarely	Sometimes	Usually	Usually	Rarely	Never	Sometimes	Always	Usually	Never	Sometimes	Rarely	Sometimes	Sometimes	Sometimes	Usually	Never	Sometimes	Sometimes	Always	Rarely	Rarely	Never	27
94	Sometimes	Rarely	Usually	Usually	Never	Never	Sometimes	Rarely	Rarely	Sometimes	Usually	Always	Never	Sometimes	Never	Usually	Rarely	Never	Usually	Never	Usually	Sometimes	Usually	Rarely	Sometimes	Usually	25
95	Always	Never	Always	Rarely	Usually	Always	Sometimes	Usually	Never	Never	Sometimes	Sometimes	Sometimes	Usually	Rarely	Sometimes	Rarely	Usually	Usually	Rarely	Usually	Sometimes	Rarely	Sometimes	Never	Never	28
96	Never	Sometimes	Always	Always	Rarely	Never	Never	Sometimes	Rarely	Sometimes	Never	Sometimes	Always	Sometimes	Sometimes	Sometimes	Rarely	Usually	Usually	Sometimes	Sometimes	Sometimes	Never	Never	Usually	Sometimes	26
97	Always	Sometimes	Always	Never	Usually	Rarely	Usually	Never	Never	Never	Always	Sometimes	Never	Sometimes	Always	Usually	Never	Never	Usually	Never	Never	Never	Rarely	Never	Usually	Never	25
98	Usually	Never	Always	Sometimes	Sometimes	Usually	Rarely	Sometimes	Never	Rarely	Rarely	Always	Sometimes	Sometimes	Rarely	Usually	Sometimes	Always	Sometimes	Sometimes	Sometimes	Sometimes	Sometimes	Rarely	Sometimes	Never	27
99	Usually	Rarely	Always	Sometimes	Never	Rarely	Rarely	Usually	Never	Sometimes	Usually	Usually	Sometimes	Usually	Usually	Rarely	Rarely	Usually	Sometimes	Sometimes	Sometimes	Sometimes	Sometimes	Sometimes	Usually	Never	28
100	Always	Sometimes	Always	Sometimes	Always	Usually	Never	Never	Never	Never	Always	Always	Never	Usually	Sometimes	Sometimes	Rarely	Rarely	Usually	Never	Sometimes	Never	Sometimes	Never	Rarely	Never	27
101	Usually	Rarely	Always	Sometimes	Sometimes	Sometimes	Usually	Sometimes	Never	Rarely	Usually	Always	Never	Usually	Sometimes	Usually	Usually	Sometimes	Usually	Sometimes	Usually	Rarely	Sometimes	Never	Sometimes	Rarely	31
102	Usually	Never	Always	Sometimes	Sometimes	Usually	Usually	Never	Never	Rarely	Sometimes	Rarely	Sometimes	Sometimes	Always	Sometimes	Rarely	Sometimes	Usually	Never	Always	Usually	Sometimes	Never	Rarely	Never	27
103	Usually	Rarely	Always	Usually	Sometimes	Usually	Never	Rarely	Never	Sometimes	Sometimes	Sometimes	Sometimes	Usually	Never	Rarely	Rarely	Sometimes	Sometimes	Usually	Usually	Usually	Rarely	Rarely	Sometimes	Never	25
104	Always	Rarely	Usually	Usually	Never	Sometimes	Sometimes	Never	Never	Usually	Always	Always	Never	Always	Sometimes	Sometimes	Rarely	Usually	Rarely	Never	Usually	Sometimes	Rarely	Never	Sometimes	Rarely	28
105	Usually	Sometimes	Sometimes	Rarely	Never	Sometimes	Usually	Never	Never	Usually	Always	Always	Never	Usually	Never	Sometimes	Rarely	Sometimes	Usually	Never	Sometimes	Sometimes	Sometimes	Rarely	Rarely	Never	24
106	Usually	Sometimes	Always	Usually	Never	Never	Rarely	Sometimes	Never	Usually	Always	Sometimes	Usually	Sometimes	Sometimes	Never	Sometimes	Sometimes	Rarely	Rarely	Sometimes	Never	Rarely	Sometimes	Always	Sometimes	27
107	Usually	Rarely	Always	Sometimes	Always	Sometimes	Sometimes	Rarely	Never	Sometimes	Usually	Sometimes	Never	Usually	Sometimes	Usually	Usually	Sometimes	Sometimes	Never	Sometimes	Sometimes	Never	Never	Rarely	Never	26
108	Always	Never	Usually	Sometimes	Sometimes	Usually	Sometimes	Sometimes	Never	Rarely	Sometimes	Usually	Sometimes	Usually	Never	Usually	Sometimes	Rarely	Sometimes	Never	Usually	Usually	Never	Sometimes	Sometimes	Never	27
109	Usually	Rarely	Always	Always	Sometimes	Sometimes	Sometimes	Sometimes	Never	Sometimes	Usually	Sometimes	Sometimes	Usually	Rarely	Sometimes	Rarely	Usually	Rarely	Rarely	Usually	Rarely	Never	Sometimes	Sometimes	Never	26
110	Always	Rarely	Always	Never	Usually	Usually	Usually	Never	Never	Sometimes	Sometimes	Rarely	Rarely	Sometimes	Rarely	Always	Usually	Sometimes	Sometimes	Never	Never	Usually	Sometimes	Never	Sometimes	Never	26
111	Sometimes	Rarely	Always	Sometimes	Always	Never	Never	Always	Never	Usually	Rarely	Never	Usually	Rarely	Rarely	Never	Never	Always	Sometimes	Always	Usually	Sometimes	Never	Never	Sometimes	Never	26
112	Always	Rarely	Usually	Sometimes	Usually	Never	Sometimes	Never	Never	Sometimes	Sometimes	Usually	Sometimes	Rarely	Usually	Sometimes	Sometimes	Sometimes	Usually	Never	Always	Sometimes	Sometimes	Sometimes	Sometimes	Never	28
113	Usually	Never	Always	Usually	Sometimes	Rarely	Sometimes	Sometimes	Never	Sometimes	Never	Sometimes	Never	Usually	Sometimes	Sometimes	Never	Usually	Sometimes	Usually	Sometimes	Sometimes	Rarely	Sometimes	Sometimes	Never	25
114	Sometimes	Sometimes	Sometimes	Sometimes	Sometimes	Usually	Sometimes	Usually	Never	Sometimes	Usually	Always	Sometimes	Sometimes	Rarely	Sometimes	Sometimes	Rarely	Usually	Rarely	Sometimes	Sometimes	Rarely	Usually	Sometimes	Never	27
115	Always	Rarely	Always	Usually	Sometimes	Always	Sometimes	Never	Never	Never	Rarely	Sometimes	Never	Sometimes	Never	Always	Usually	Never	Usually	Never	Rarely	Never	Rarely	Never	Rarely	Never	22

116	Sometimes	Rarely	Usually	Never	Always	Usually	Usually	Rarely	Never	Sometimes	Sometimes	Usually	Never	Sometimes	Always	Sometimes	Never	Always	Usually	Never	Rarely	Never	Rarely	Never	Rarely	Never	24
117	Usually	Rarely	Always	Never	Sometimes	Rarely	Usually	Never	Never	Rarely	Always	Always	Never	Usually	Sometimes	Usually	Never	Usually	Usually	Never	Rarely	Rarely	Rarely	Rarely	Never	Never	23
118	Usually	Sometimes	Always	Rarely	Never	Sometimes	Sometimes	Sometimes	Never	Sometimes	Usually	Sometimes	Sometimes	Usually	Sometimes	Usually	Rarely	Sometimes	Always	Sometimes	Rarely	Rarely	Never	Never	Sometimes	Never	25
119	Never	Rarely	Rarely	Rarely	Always	Usually	Sometimes	Sometimes	Never	Rarely	Rarely	Sometimes	Sometimes	Sometimes	Never	Usually	Sometimes	Sometimes	Usually	Sometimes	Usually	Always	Never	Never	Sometimes	Never	23
120	Always	Rarely	Rarely	Always	Sometimes	Rarely	Rarely	Never	Never	Sometimes	Always	Always	Never	Always	Never	Rarely	Rarely	Sometimes	Rarely	Never	Usually	Never	Sometimes	Never	Usually	Never	23
121	Usually	Rarely	Always	Usually	Sometimes	Rarely	Sometimes	Usually	Never	Rarely	Usually	Usually	Sometimes	Usually	Sometimes	Sometimes	Rarely	Usually	Sometimes	Rarely	Sometimes	Rarely	Rarely	Never	Rarely	Never	24
122	Usually	Usually	Always	Usually	Sometimes	Sometimes	Rarely	Rarely	Never	Sometimes	Usually	Usually	Never	Rarely	Rarely	Rarely	Rarely	Usually	Rarely	Rarely	Usually	Sometimes	Usually	Rarely	Always	Rarely	26
123	Usually	Sometimes	Sometimes	Sometimes	Sometimes	Sometimes	Usually	Sometimes	Never	Never	Sometimes	Sometimes	Sometimes	Usually	Never	Never	Sometimes	Sometimes	Sometimes	Sometimes	Sometimes	Never	Sometimes	Never	Sometimes	Never	22
124	Never	Never	Always	Rarely	Sometimes	Never	Never	Always	Never	Never	Never	Never	Usually	Never	Usually	Sometimes	Rarely	Always	Sometimes	Usually	Usually	Sometimes	Never	Never	Usually	Rarely	23
125	Sometimes	Never	Always	Never	Never	Usually	Sometimes	Sometimes	Never	Sometimes	Never	Never	Sometimes	Always	Rarely	Sometimes	Sometimes	Always	Always	Sometimes	Sometimes	Sometimes	Never	Sometimes	Sometimes	Never	26
126	Always	Rarely	Sometimes	Always	Sometimes	Rarely	Rarely	Sometimes	Never	Always	Usually	Rarely	Rarely	Sometimes	Always	Rarely	Rarely	Usually	Rarely	Sometimes	Sometimes	Always	Rarely	Never	Sometimes	Rarely	26
127	Sometimes	Never	Usually	Sometimes	Sometimes	Always	Rarely	Never	Never	Rarely	Always	Always	Never	Never	Usually	Never	Never	Rarely	Always	Never	Sometimes	Never	Sometimes	Sometimes	Always	Never	25
128	Sometimes	Sometimes	Always	Rarely	Sometimes	Usually	Sometimes	Sometimes	Never	Sometimes	Sometimes	Sometimes	Sometimes	Usually	Sometimes	Usually	Sometimes	Usually	Usually	Sometimes	Sometimes	Sometimes	Sometimes	Sometimes	Sometimes	Never	30
129	Usually	Usually	Always	Usually	Sometimes	Rarely	Sometimes	Rarely	Never	Rarely	Usually	Always	Never	Usually	Rarely	Rarely	Never	Sometimes	Sometimes	Sometimes	Sometimes	Never	Sometimes	Rarely	Usually	Never	25
130	Always	Rarely	Always	Sometimes	Always	Rarely	Rarely	Never	Never	Sometimes	Sometimes	Rarely	Never	Usually	Always	Rarely	Sometimes	Rarely	Sometimes	Never	Usually	Sometimes	Usually	Rarely	Sometimes	Never	25
131	Always	Sometimes	Always	Sometimes	Sometimes	Rarely	Rarely	Sometimes	Never	Rarely	Always	Sometimes	Rarely	Always	Sometimes	Sometimes	Never	Sometimes	Usually	Usually	Sometimes	Usually	Rarely	Rarely	Sometimes	Rarely	28
132	Always	Rarely	Usually	Usually	Usually	Usually	Rarely	Rarely	Never	Usually	Always	Sometimes	Never	Usually	Sometimes	Sometimes	Rarely	Sometimes	Sometimes	Rarely	Sometimes	Rarely	Rarely	Never	Sometimes	Never	25
133	Always	Never	Usually	Sometimes	Sometimes	Never	Never	Never	Never	Sometimes	Always	Sometimes	Never	Usually	Usually	Never	Rarely	Rarely	Sometimes	Rarely	Always	Sometimes	Sometimes	Never	Sometimes	Sometimes	24
134	Sometimes	Rarely	Usually	Sometimes	Usually	Sometimes	Sometimes	Usually	Never	Rarely	Sometimes	Rarely	Rarely	Sometimes	Usually	Rarely	Rarely	Usually	Always	Usually	Rarely	Rarely	Sometimes	Never	Never	Never	22
135	Always	Rarely	Usually	Usually	Sometimes	Sometimes	Never	Never	Never	Usually	Always	Sometimes	Never	Usually	Sometimes	Never	Sometimes	Usually	Sometimes	Never	Rarely	Sometimes	Rarely	Rarely	Sometimes	Never	24
136	Usually	Sometimes	Usually	Sometimes	Usually	Sometimes	Never	Never	Never	Sometimes	Usually	Usually	Never	Usually	Never	Sometimes	Sometimes	Sometimes	Sometimes	Never	Usually	Never	Usually	Never	Never	Never	24
137	Usually	Never	Always	Usually	Rarely	Rarely	Sometimes	Never	Never	Sometimes	Sometimes	Usually	Never	Sometimes	Sometimes	Sometimes	Sometimes	Always	Usually	Usually	Sometimes	Sometimes	Rarely	Never	Rarely	Never	25

138	Rarely	Never	Always	Never	Sometimes	Never	Never	Always	Never	Never	Rarely	Never	Always	Rarely	Sometimes	Never	Never	Always	Sometimes	Usually	Usually	Never	Never	Never	Always	Never	22
139	Always	Sometimes	Usually	Sometimes	Sometimes	Never	Sometimes	Never	Never	Sometimes	Usually	Usually	Never	Usually	Sometimes	Rarely	Sometimes	Sometimes	Rarely	Never	Usually	Never	Sometimes	Never	Usually	Never	24
140	Always	Sometimes	Usually	Sometimes	Usually	Never	Rarely	Never	Never	Sometimes	Sometimes	Sometimes	Never	Sometimes	Always	Sometimes	Rarely	Sometimes	Usually	Rarely	Sometimes	Usually	Rarely	Never	Sometimes	Never	24
141	Usually	Rarely	Always	Sometimes	Usually	Never	Rarely	Sometimes	Never	Sometimes	Usually	Rarely	Sometimes	Usually	Sometimes	Sometimes	Never	Rarely	Usually	Sometimes	Sometimes	Sometimes	Usually	Never	Never	Never	24
142	Usually	Rarely	Usually	Sometimes	Rarely	Sometimes	Sometimes	Never	Never	Sometimes	Usually	Sometimes	Never	Usually	Never	Sometimes	Rarely	Sometimes	Sometimes	Never	Sometimes	Sometimes	Usually	Sometimes	Sometimes	Never	22
143	Sometimes	Sometimes	Always	Sometimes	Sometimes	Rarely	Rarely	Sometimes	Never	Sometimes	Usually	Usually	Never	Sometimes	Sometimes	Rarely	Sometimes	Usually	Sometimes	Sometimes	Usually	Never	Never	Never	Sometimes	Never	23
144	Always	Rarely	Sometimes	Sometimes	Never	Usually	Sometimes	Rarely	Never	Usually	Usually	Sometimes	Never	Sometimes	Rarely	Sometimes	Rarely	Sometimes	Usually	Never	Always	Never	Usually	Rarely	Rarely	Never	23
145	Sometimes	Never	Always	Never	Never	Never	Rarely	Sometimes	Never	Never	Sometimes	Usually	Sometimes	Sometimes	Sometimes	Never	Never	Always	Usually	Sometimes	Sometimes	Rarely	Rarely	Never	Always	Never	21
146	Sometimes	Sometimes	Always	Rarely	Usually	Usually	Sometimes	Sometimes	Never	Sometimes	Sometimes	Rarely	Rarely	Sometimes	Always	Sometimes	Sometimes	Sometimes	Usually	Sometimes	Sometimes	Usually	Rarely	Never	Sometimes	Never	27
147	Always	Rarely	Always	Sometimes	Sometimes	Sometimes	Sometimes	Sometimes	Never	Rarely	Sometimes	Sometimes	Sometimes	Sometimes	Sometimes	Usually	Rarely	Usually	Usually	Rarely	Usually	Usually	Rarely	Sometimes	Rarely	Never	27
148	Sometimes	Rarely	Usually	Sometimes	Always	Rarely	Sometimes	Rarely	Never	Never	Rarely	Sometimes	Usually	Sometimes	Sometimes	Sometimes	Rarely	Sometimes	Always	Sometimes	Sometimes	Sometimes	Never	Sometimes	Sometimes	Sometimes	24
149	Sometimes	Never	Always	Never	Usually	Sometimes	Sometimes	Always	Never	Rarely	Sometimes	Usually	Never	Sometimes	Sometimes	Sometimes	Sometimes	Usually	Usually	Sometimes	Never	Rarely	Rarely	Never	Sometimes	Never	24
150	Always	Rarely	Sometimes	Sometimes	Usually	Always	Never	Never	Never	Rarely	Sometimes	Always	Never	Sometimes	Rarely	Never	Never	Never	Always	Sometimes	Sometimes	Never	Sometimes	Sometimes	Sometimes	Never	23
151	Never	Sometimes	Usually	Never	Never	Never	Never	Always	Never	Sometimes	Never	Never	Usually	Never	Usually	Sometimes	Sometimes	Sometimes	Sometimes	Always	Sometimes	Sometimes	Never	Never	Usually	Sometimes	23
152	Usually	Rarely	Usually	Sometimes	Usually	Never	Rarely	Rarely	Never	Sometimes	Sometimes	Sometimes	Rarely	Sometimes	Usually	Usually	Sometimes	Rarely	Usually	Sometimes	Usually	Sometimes	Rarely	Rarely	Usually	Never	24
153	Sometimes	Sometimes	Always	Rarely	Usually	Never	Never	Usually	Never	Rarely	Sometimes	Never	Usually	Sometimes	Sometimes	Usually	Rarely	Rarely	Usually	Usually	Rarely	Sometimes	Sometimes	Rarely	Sometimes	Never	23
154	Always	Sometimes	Usually	Sometimes	Never	Never	Never	Never	Never	Always	Always	Rarely	Never	Always	Sometimes	Never	Never	Sometimes	Never	Never	Always	Sometimes	Rarely	Never	Sometimes	Never	23
155	Always	Rarely	Usually	Sometimes	Never	Usually	Usually	Never	Never	Sometimes	Usually	Usually	Rarely	Usually	Rarely	Sometimes	Sometimes	Sometimes	Sometimes	Never	Sometimes	Rarely	Sometimes	Never	Sometimes	Never	24
156	Sometimes	Never	Always	Never	Usually	Never	Always	Sometimes	Never	Never	Never	Never	Always	Never	Never	Always	Never	Never	Always	Never	Never	Usually	Never	Never	Never	Never	21
157	Usually	Sometimes	Always	Sometimes	Sometimes	Rarely	Sometimes	Rarely	Never	Sometimes	Always	Always	Sometimes	Usually	Sometimes	Rarely	Sometimes	Sometimes	Sometimes	Sometimes	Sometimes	Sometimes	Sometimes	Never	Sometimes	Never	28
158	Sometimes	Sometimes	Sometimes	Sometimes	Sometimes	Sometimes	Sometimes	Usually	Sometimes	Rarely	Rarely	Sometimes	Usually	Sometimes	Sometimes	Usually	Sometimes	Sometimes	Always	Usually	Rarely	Sometimes	Never	Sometimes	Sometimes	Sometimes	28
159	Always	Rarely	Sometimes	Sometimes	Sometimes	Sometimes	Sometimes	Sometimes	Never	Sometimes	Always	Usually	Sometimes	Always	Rarely	Sometimes	Some	Sometimes	Sometimes	Sometimes	Sometimes	Sometimes	Sometimes	Rarely	Rarely	Rarely	27

																times											
160	Sometimes	Rarely	Always	Rarely	Rarely	Sometimes	Never	Rarely	Never	Rarely	Rarely	Rarely	Rarely	Rarely	Sometimes	Usually	Usually	Usually	Usually	Usually	Sometimes	Sometimes	Rarely	Never	Sometimes	Never	19
161	Never	Rarely	Sometimes	Never	Always	Usually	Usually	Sometimes	Never	Never	Never	Rarely	Usually	Sometimes	Always	Sometimes	Rarely	Sometimes	Always	Sometimes	Sometimes	Rarely	Never	Sometimes	Sometimes	Never	24
162	Sometimes	Never	Always	Never	Always	Sometimes	Never	Rarely	Never	Rarely	Rarely	Sometimes	Never	Sometimes	Always	Sometimes	Never	Rarely	Usually	Never	Usually	Never	Rarely	Never	Sometimes	Never	19
163	Sometimes	Never	Usually	Never	Sometimes	Sometimes	Never	Usually	Never	Never	Never	Sometimes	Always	Usually	Never	Never	Sometimes	Sometimes	Always	Usually	Sometimes	Never	Never	Never	Sometimes	Never	22
164	Never	Never	Usually	Sometimes	Always	Sometimes	Rarely	Usually	Never	Never	Never	Sometimes	Usually	Usually	Never	Sometimes	Usually	Sometimes	Usually	Sometimes	Rarely	Never	Never	Never	Sometimes	Never	22
165	Usually	Sometimes	Always	Never	Usually	Sometimes	Sometimes	Never	Never	Never	Sometimes	Sometimes	Never	Usually	Usually	Usually	Usually	Never	Usually	Never	Rarely	Never	Rarely	Never	Never	Never	22
166	Always	Sometimes	Always	Never	Never	Never	Never	Always	Never	Sometimes	Always	Sometimes	Sometimes	Sometimes	Rarely	Sometimes	Never	Sometimes	Sometimes	Sometimes	Sometimes	Rarely	Sometimes	Never	Sometimes	Never	24
167	Never	Never	Always	Rarely	Never	Never	Never	Sometimes	Never	Never	Never	Rarely	Always	Never	Rarely	Sometimes	Never	Rarely	Always	Always	Sometimes	Always	Never	Never	Usually	Usually	22
168	Usually	Sometimes	Always	Sometimes	Usually	Sometimes	Sometimes	Rarely	Never	Sometimes	Sometimes	Always	Rarely	Usually	Rarely	Sometimes	Sometimes	Rarely	Usually	Rarely	Rarely	Sometimes	Sometimes	Never	Sometimes	Never	25
169	Sometimes	Rarely	Usually	Never	Sometimes	Sometimes	Rarely	Always	Never	Never	Sometimes	Never	Sometimes	Rarely	Always	Sometimes	Rarely	Never	Always	Sometimes	Rarely	Sometimes	Never	Never	Sometimes	Never	20
170	Always	Sometimes	Sometimes	Never	Rarely	Rarely	Never	Usually	Never	Sometimes	Always	Always	Sometimes	Always	Rarely	Rarely	Sometimes	Rarely	Usually	Sometimes	Rarely	Sometimes	Never	Sometimes	Sometimes	Never	25
171	Never	Sometimes	Sometimes	Rarely	Rarely	Sometimes	Rarely	Usually	Rarely	Sometimes	Never	Never	Sometimes	Never	Usually	Sometimes	Never	Always	Sometimes	Always	Usually	Sometimes	Never	Sometimes	Usually	Usually	25
172	Rarely	Never	Always	Usually	Never	Never	Never	Never	Never	Never	Never	Never	Never	Never	Always	Rarely	Never	Always	Usually	Rarely	Always	Never	Never	Never	Always	Never	19
173	Usually	Rarely	Sometimes	Sometimes	Sometimes	Sometimes	Sometimes	Never	Never	Sometimes	Always	Usually	Never	Usually	Sometimes	Rarely	Sometimes	Usually	Usually	Never	Rarely	Sometimes	Sometimes	Sometimes	Never	Rarely	24
174	Always	Sometimes	Usually	Usually	Sometimes	Rarely	Rarely	Rarely	Never	Sometimes	Usually	Sometimes	Never	Always	Sometimes	Rarely	Rarely	Sometimes	Sometimes	Rarely	Sometimes	Sometimes	Rarely	Never	Sometimes	Rarely	22
175	Never	Never	Always	Never	Sometimes	Never	Always	Never	Never	Never	Never	Never	Sometimes	Never	Never	Always	Rarely	Sometimes	Usually	Never	Usually	Never	Never	Sometimes	Usually	Never	19
176	Usually	Never	Sometimes	Sometimes	Usually	Sometimes	Rarely	Never	Never	Sometimes	Sometimes	Sometimes	Rarely	Sometimes	Usually	Rarely	Sometimes	Sometimes	Sometimes	Rarely	Usually	Sometimes	Sometimes	Never	Sometimes	Sometimes	22
177	Sometimes	Never	Always	Never	Sometimes	Never	Never	Always	Never	Never	Sometimes	Never	Sometimes	Sometimes	Sometimes	Sometimes	Never	Never	Always	Sometimes	Never	Never	Never	Sometimes	Never	Never	18
178	Sometimes	Never	Usually	Never	Rarely	Sometimes	Sometimes	Usually	Never	Sometimes	Rarely	Sometimes	Usually	Sometimes	Sometimes	Sometimes	Never	Rarely	Usually	Sometimes	Sometimes	Sometimes	Sometimes	Never	Sometimes	Never	21
179	Never	Never	Always	Never	Always	Rarely	Never	Always	Never	Never	Never	Never	Always	Rarely	Sometimes	Never	Never	Never	Usually	Sometimes	Sometimes	Never	Never	Never	Always	Never	20
180	Always	Never	Always	Never	Usually	Rarely	Sometimes	Never	Never	Never	Sometimes	Sometimes	Never	Sometimes	Usually	Usually	Never	Never	Always	Never	Rarely	Sometimes	Never	Never	Never	Never	20
181	Sometimes	Rarely	Always	Never	Usually	Sometimes	Usually	Sometimes	Never	Rarely	Sometimes	Never	Sometimes	Never	Never	Always	Sometimes	Never	Usually	Never	Never	Never	Never	Never	Never	Never	18

182	Sometimes	Rarely	Always	Rarely	Always	Always	Rarely	Sometimes	Never	Never	Rarely	Rarely	Never	Rarely	Rarely	Usually	Sometimes	Never	Usually	Rarely	Sometimes	Sometimes	Rarely	Rarely	Rarely	Never	18
183	Rarely	Never	Always	Never	Usually	Never	Rarely	Never	Rarely	Sometimes	Sometimes	Always	Never	Always	Never	Never	Never	Sometimes	Rarely	Rarely	Always	Always	Rarely	Never	Rarely	Never	20
184	Sometimes	Sometimes	Usually	Usually	Sometimes	Never	Rarely	Sometimes	Never	Usually	Sometimes	Rarely	Sometimes	Usually	Sometimes	Rarely	Rarely	Usually	Rarely	Sometimes	Sometimes	Sometimes	Never	Never	Sometimes	Never	21
185	Sometimes	Never	Always	Never	Sometimes	Usually	Sometimes	Rarely	Never	Sometimes	Usually	Rarely	Never	Always	Never	Sometimes	Sometimes	Sometimes	Sometimes	Rarely	Sometimes	Sometimes	Usually	Never	Never	Never	22
186	Usually	Never	Usually	Sometimes	Sometimes	Never	Never	Rarely	Never	Sometimes	Always	Always	Never	Sometimes	Sometimes	Never	Sometimes	Never	Sometimes	Rarely	Usually	Usually	Never	Never	Always	Never	24
187	Never	Never	Always	Never	Usually	Never	Rarely	Usually	Never	Never	Never	Never	Sometimes	Rarely	Always	Rarely	Never	Sometimes	Always	Never	Sometimes	Sometimes	Never	Never	Sometimes	Never	18
188	Always	Sometimes	Usually	Sometimes	Sometimes	Never	Never	Rarely	Never	Rarely	Usually	Sometimes	Sometimes	Usually	Rarely	Rarely	Sometimes	Usually	Sometimes	Rarely	Usually	Never	Never	Never	Sometimes	Never	21
189	Always	Usually	Always	Sometimes	Sometimes	Never	Never	Usually	Never	Sometimes	Sometimes	Never	Rarely	Sometimes	Rarely	Never	Never	Sometimes	Sometimes	Sometimes	Sometimes	Never	Sometimes	Sometimes	Sometimes	Never	22
190	Sometimes	Never	Usually	Sometimes	Usually	Never	Never	Never	Never	Never	Sometimes	Never	Usually	Sometimes	Always	Sometimes	Sometimes	Usually	Usually	Never	Usually	Sometimes	Never	Never	Rarely	Never	22
191	Never	Rarely	Usually	Never	Usually	Usually	Sometimes	Never	Never	Never	Sometimes	Never	Rarely	Rarely	Usually	Sometimes	Sometimes	Never	Always	Sometimes	Never	Rarely	Rarely	Usually	Rarely	Never	18
192	Usually	Never	Never	Sometimes	Usually	Usually	Usually	Rarely	Never	Never	Sometimes	Usually	Never	Usually	Sometimes	Sometimes	Rarely	Never	Usually	Rarely	Rarely	Rarely	Sometimes	Sometimes	Rarely	Rarely	20
193	Always	Sometimes	Always	Sometimes	Usually	Sometimes	Sometimes	Sometimes	Never	Rarely	Sometimes	Rarely	Never	Sometimes	Always	Sometimes	Sometimes	Usually	Usually	Rarely	Rarely	Rarely	Never	Never	Never	Never	24
194	Sometimes	Rarely	Usually	Sometimes	Sometimes	Rarely	Rarely	Usually	Never	Never	Sometimes	Usually	Never	Sometimes	Rarely	Sometimes	Sometimes	Usually	Sometimes	Sometimes	Sometimes	Sometimes	Sometimes	Rarely	Sometimes	Never	21
195	Usually	Sometimes	Sometimes	Rarely	Rarely	Rarely	Rarely	Always	Never	Sometimes	Usually	Usually	Rarely	Usually	Never	Rarely	Never	Sometimes	Sometimes	Sometimes	Sometimes	Rarely	Sometimes	Sometimes	Sometimes	Never	21
196	Usually	Sometimes	Always	Sometimes	Usually	Rarely	Rarely	Never	Never	Sometimes	Always	Sometimes	Never	Rarely	Sometimes	Sometimes	Rarely	Sometimes	Sometimes	Rarely	Usually	Sometimes	Sometimes	Sometimes	Sometimes	Never	24
197	Usually	Sometimes	Usually	Sometimes	Rarely	Usually	Sometimes	Rarely	Never	Rarely	Sometimes	Sometimes	Sometimes	Usually	Sometimes	Usually	Rarely	Never	Usually	Rarely	Rarely	Sometimes	Never	Sometimes	Sometimes	Never	22
198	Always	Rarely	Always	Sometimes	Never	Never	Always	Never	Rarely	Never	Sometimes	Rarely	Never	Sometimes	Usually	Never	Never	Never	Never	Sometimes	Sometimes	Never	Rarely	Never	Sometimes	Sometimes	18
199	Usually	Sometimes	Usually	Sometimes	Always	Sometimes	Rarely	Sometimes	Never	Rarely	Always	Never	Sometimes	Always	Rarely	Sometimes	Rarely	Never	Usually	Rarely	Rarely	Sometimes	Never	Never	Never	Never	22
200	Always	Never	Always	Rarely	Always	Sometimes	Sometimes	Never	Never	Rarely	Sometimes	Rarely	Rarely	Rarely	Usually	Sometimes	Rarely	Rarely	Usually	Sometimes	Sometimes	Rarely	Sometimes	Rarely	Sometimes	Never	21
201	Usually	Never	Usually	Rarely	Sometimes	Usually	Sometimes	Never	Never	Sometimes	Sometimes	Sometimes	Never	Sometimes	Sometimes	Rarely	Sometimes	Sometimes	Usually	Rarely	Sometimes	Sometimes	Never	Never	Usually	Never	21
202	Usually	Usually	Always	Sometimes	Sometimes	Never	Never	Never	Never	Never	Usually	Rarely	Never	Sometimes	Rarely	Never	Sometimes	Never	Sometimes	Never	Usually	Never	Never	Sometimes	Sometimes	Never	18

203	Usually	Sometimes	Always	Usually	Sometimes	Never	Usually	Rarely	Never	Sometimes	Sometimes	Never	Never	Never	Never	Never	Usually	Usually	Sometimes	Sometimes	Usually	Never	Never	Never	Never	Never	21
204	Sometimes	Rarely	Sometimes	Rarely	Always	Sometimes	Rarely	Never	Never	Rarely	Always	Rarely	Never	Sometimes	Never	Rarely	Sometimes	Never	Usually	Never	Sometimes	Never	Sometimes	Never	Usually	Never	17
205	Usually	Sometimes	Usually	Sometimes	Usually	Sometimes	Sometimes	Rarely	Never	Rarely	Rarely	Rarely	Never	Always	Rarely	Sometimes	Sometimes	Rarely	Sometimes	Rarely	Sometimes	Sometimes	Sometimes	Sometimes	Sometimes	Never	21
206	Always	Rarely	Sometimes	Always	Sometimes	Never	Never	Never	Never	Sometimes	Sometimes	Rarely	Sometimes	Sometimes	Never	Never	Never	Sometimes	Usually	Never	Always	Never	Sometimes	Never	Usually	Never	21
207	Sometimes	Never	Always	Sometimes	Never	Rarely	Never	Sometimes	Never	Sometimes	Rarely	Never	Never	Always	Never	Sometimes	Never	Sometimes	Rarely	Sometimes	Usually	Sometimes	Sometimes	Never	Never	Never	17
208	Sometimes	Rarely	Always	Rarely	Always	Sometimes	Sometimes	Never	Never	Never	Rarely	Usually	Rarely	Sometimes	Usually	Sometimes	Rarely	Rarely	Sometimes	Never	Rarely	Sometimes	Rarely	Never	Never	Never	17
209	Sometimes	Usually	Always	Rarely	Sometimes	Never	Never	Sometimes	Never	Never	Rarely	Never	Rarely	Rarely	Usually	Sometimes	Never	Usually	Always	Rarely	Never	Sometimes	Never	Sometimes	Rarely	Never	18
210	Sometimes	Rarely	Always	Usually	Sometimes	Never	Sometimes	Sometimes	Never	Rarely	Rarely	Usually	Rarely	Sometimes	Never	Rarely	Rarely	Sometimes	Sometimes	Sometimes	Sometimes	Sometimes	Never	Sometimes	Sometimes	Never	19
211	Rarely	Never	Usually	Usually	Sometimes	Rarely	Never	Rarely	Never	Rarely	Rarely	Sometimes	Sometimes	Rarely	Usually	Sometimes	Sometimes	Rarely	Always	Rarely	Usually	Rarely	Never	Sometimes	Sometimes	Never	18
212	Sometimes	Rarely	Usually	Never	Sometimes	Usually	Rarely	Never	Never	Sometimes	Sometimes	Always	Sometimes	Usually	Rarely	Sometimes	Rarely	Never	Usually	Never	Sometimes	Rarely	Sometimes	Never	Sometimes	Never	20
213	Sometimes	Never	Usually	Never	Usually	Sometimes	Never	Never	Never	Usually	Usually	Sometimes	Rarely	Sometimes	Never	Never	Rarely	Always	Sometimes	Never	Sometimes	Never	Rarely	Never	Sometimes	Never	18
214	Sometimes	Rarely	Always	Rarely	Never	Sometimes	Never	Never	Never	Never	Sometimes	Usually	Sometimes	Sometimes	Sometimes	Never	Never	Usually	Always	Sometimes	Sometimes	Never	Rarely	Never	Sometimes	Never	19
215	Sometimes	Never	Always	Sometimes	Never	Never	Never	Never	Never	Sometimes	Sometimes	Sometimes	Never	Always	Sometimes	Sometimes	Never	Sometimes	Sometimes	Never	Rarely	Sometimes	Never	Never	Never	Never	16
216	Rarely	Sometimes	Always	Sometimes	Sometimes	Rarely	Rarely	Sometimes	Rarely	Never	Never	Never	Sometimes	Never	Usually	Rarely	Rarely	Always	Sometimes	Usually	Sometimes	Rarely	Rarely	Rarely	Sometimes	Rarely	18
217	Sometimes	Never	Always	Never	Always	Never	Never	Sometimes	Never	Never	Never	Sometimes	Sometimes	Sometimes	Sometimes	Usually	Sometimes	Never	Usually	Never	Never	Never	Never	Never	Usually	Never	19
218	Always	Rarely	Always	Sometimes	Sometimes	Never	Usually	Rarely	Never	Rarely	Never	Rarely	Sometimes	Rarely	Rarely	Rarely	Rarely	Always	Sometimes	Rarely	Sometimes	Rarely	Never	Never	Sometimes	Never	17
219	Rarely	Never	Always	Never	Sometimes	Never	Never	Sometimes	Never	Never	Never	Never	Usually	Never	Rarely	Sometimes	Sometimes	Usually	Usually	Never	Rarely	Sometimes	Never	Never	Always	Never	17
220	Never	Rarely	Usually	Never	Usually	Usually	Never	Sometimes	Rarely	Never	Never	Never	Sometimes	Never	Usually	Sometimes	Sometimes	Sometimes	Always	Usually	Sometimes	Rarely	Never	Never	Rarely	Never	19
221	Usually	Rarely	Usually	Sometimes	Usually	Never	Rarely	Never	Never	Sometimes	Sometimes	Never	Never	Always	Never	Usually	Rarely	Rarely	Sometimes	Never	Sometimes	Never	Sometimes	Never	Never	Never	17
222	Sometimes	Sometimes	Usually	Never	Sometimes	Sometimes	Sometimes	Never	Never	Never	Sometimes	Rarely	Sometimes	Sometimes	Always	Sometimes	Sometimes	Usually	Usually	Rarely	Rarely	Sometimes	Sometimes	Rarely	Rarely	Sometimes	22
223	Never	Never	Always	Never	Never	Never	Never	Always	Never	Sometimes	Never	Never	Always	Never	Sometimes	Never	Never	Sometimes	Usually	Always	Sometimes	Never	Never	Never	Never	Never	18
224	Rarely	Rarely	Usually	Rarely	Always	Rarely	Never	Sometimes	Never	Never	Never	Rarely	Sometimes	Sometimes	Never	Rarely	Always	Always	Always	Sometimes	Rarely	Rarely	Rarely	Never	Sometimes	Never	19

225	Sometimes	Rarely	Sometimes	Rarely	Sometimes	Sometimes	Rarely	Never	Never	Sometimes	Sometimes	Rarely	Never	Sometimes	Sometimes	Sometimes	Rarely	Sometimes	Usually	Sometimes	Sometimes	Sometimes	Usually	Rarely	Sometimes	Never	18
226	Usually	Sometimes	Always	Usually	Never	Never	Never	Rarely	Never	Rarely	Usually	Sometimes	Sometimes	Sometimes	Sometimes	Rarely	Never	Sometimes	Sometimes	Sometimes	Rarely	Sometimes	Sometimes	Rarely	Rarely	Never	19
227	Sometimes	Never	Usually	Never	Never	Sometimes	Never	Never	Never	Never	Never	Sometimes	Never	Usually	Sometimes	Never	Rarely	Sometimes	Usually	Never	Usually	Sometimes	Never	Never	Usually	Never	16
228	Always	Sometimes	Sometimes	Rarely	Always	Rarely	Rarely	Rarely	Never	Usually	Sometimes	Sometimes	Rarely	Usually	Sometimes	Sometimes	Never	Rarely	Rarely	Never	Sometimes	Sometimes	Rarely	Sometimes	Sometimes	Never	20
229	Always	Never	Always	Never	Sometimes	Always	Never	Never	Never	Never	Never	Never	Sometimes	Usually	Sometimes	Sometimes	Sometimes	Never	Usually	Never	Sometimes	Never	Never	Never	Never	Never	19
230	Sometimes	Rarely	Always	Never	Sometimes	Never	Never	Rarely	Never	Never	Sometimes	Sometimes	Never	Rarely	Always	Never	Never	Usually	Usually	Never	Sometimes	Never	Rarely	Never	Sometimes	Never	16
231	Usually	Rarely	Sometimes	Sometimes	Sometimes	Never	Rarely	Never	Never	Rarely	Usually	Sometimes	Never	Always	Sometimes	Never	Never	Sometimes	Rarely	Rarely	Usually	Rarely	Rarely	Never	Usually	Never	17
232	Sometimes	Sometimes	Sometimes	Usually	Sometimes	Never	Never	Never	Never	Sometimes	Never	Always	Never	Sometimes	Rarely	Sometimes	Never	Never	Usually	Never	Rarely	Sometimes	Rarely	Sometimes	Sometimes	Never	17
233	Sometimes	Sometimes	Usually	Never	Never	Never	Never	Never	Never	Sometimes	Never	Never	Never	Sometimes	Always	Never	Never	Never	Usually	Sometimes	Rarely	Never	Sometimes	Never	Usually	Sometimes	16
234	Rarely	Rarely	Sometimes	Never	Rarely	Rarely	Sometimes	Rarely	Never	Sometimes	Sometimes	Sometimes	Never	Never	Sometimes	Usually	Sometimes	Never	Always	Sometimes	Rarely	Sometimes	Never	Sometimes	Never	Never	15
235	Sometimes	Rarely	Usually	Sometimes	Sometimes	Usually	Sometimes	Never	Never	Rarely	Rarely	Rarely	Rarely	Rarely	Sometimes	Sometimes	Rarely	Usually	Always	Sometimes	Usually	Sometimes	Rarely	Never	Sometimes	Never	20
236	Sometimes	Sometimes	Usually	Sometimes	Sometimes	Sometimes	Sometimes	Sometimes	Rarely	Rarely	Sometimes	Sometimes	Sometimes	Usually	Sometimes	Usually	Sometimes	Sometimes	Usually	Sometimes	Sometimes	Sometimes	Sometimes	Sometimes	Sometimes	Rarely	27
237	Sometimes	Never	Usually	Sometimes	Usually	Sometimes	Never	Never	Never	Never	Usually	Rarely	Never	Sometimes	Sometimes	Never	Never	Always	Sometimes	Never	Sometimes	Sometimes	Sometimes	Never	Sometimes	Never	19
238	Rarely	Never	Usually	Sometimes	Always	Never	Never	Never	Never	Sometimes	Rarely	Rarely	Sometimes	Usually	Always	Never	Rarely	Sometimes	Sometimes	Never	Usually	Sometimes	Never	Never	Rarely	Never	18
239	Usually	Rarely	Always	Sometimes	Never	Rarely	Rarely	Rarely	Never	Sometimes	Usually	Usually	Never	Sometimes	Always	Sometimes	Never	Never	Sometimes	Rarely	Sometimes	Sometimes	Never	Never	Sometimes	Never	20
240	Always	Never	Always	Never	Always	Never	Sometimes	Never	Never	Never	Rarely	Never	Rarely	Rarely	Never	Never	Never	Always	Always	Sometimes	Rarely	Never	Never	Never	Rarely	Never	17
241	Never	Never	Never	Never	Sometimes	Never	Never	Usually	Never	Never	Never	Never	Always	Never	Usually	Never	Sometimes	Sometimes	Sometimes	Usually	Sometimes	Never	Never	Never	Usually	Never	16
242	Sometimes	Never	Always	Usually	Always	Rarely	Never	Never	Never	Rarely	Never	Never	Rarely	Never	Sometimes	Rarely	Never	Never	Usually	Never	Sometimes	Sometimes	Never	Rarely	Sometimes	Rarely	15
243	Rarely	Sometimes	Sometimes	Never	Usually	Never	Rarely	Usually	Never	Never	Never	Never	Usually	Never	Rarely	Rarely	Sometimes	Sometimes	Usually	Sometimes	Usually	Never	Never	Never	Sometimes	Never	16
244	Sometimes	Never	Always	Never	Rarely	Never	Never	Usually	Never	Never	Never	Never	Usually	Never	Never	Sometimes	Sometimes	Never	Always	Always	Never	Never	Never	Never	Sometimes	Never	17
245	Sometimes	Rarely	Usually	Rarely	Usually	Sometimes	Sometimes	Rarely	Never	Rarely	Sometimes	Sometimes	Rarely	Sometimes	Always	Sometimes	Sometimes	Rarely	Always	Rarely	Rarely	Rarely	Rarely	Rarely	Sometimes	Never	19
246	Usually	Sometimes	Usually	Sometimes	Sometimes	Rarely	Never	Never	Never	Never	Sometimes	Usually	Never	Sometimes	Sometimes	Never	Rarely	Never	Always	Never	Rarely	Never	Never	Never	Sometimes	Never	16

247	Never	Rarely	Always	Rarely	Sometimes	Sometimes	Sometimes	Sometimes	Never	Rarely	Never	Rarely	Always	Never	Sometimes	Sometimes	Rarely	Sometimes	Sometimes	Sometimes	Usually	Sometimes	Never	Never	Sometimes	Never	19
248	Never	Sometimes	Usually	Never	Usually	Rarely	Rarely	Sometimes	Rarely	Rarely	Never	Never	Usually	Sometimes	Usually	Sometimes	Rarely	Sometimes	Usually	Rarely	Sometimes	Never	Never	Never	Sometimes	Rarely	17
249	Sometimes	Rarely	Usually	Sometimes	Sometimes	Sometimes	Sometimes	Sometimes	Never	Sometimes	Rarely	Rarely	Never	Rarely	Rarely	Sometimes	Sometimes	Sometimes	Usually	Sometimes	Usually	Sometimes	Never	Never	Usually	Never	20
250	Rarely	Never	Always	Never	Sometimes	Sometimes	Sometimes	Rarely	Never	Never	Rarely	Never	Never	Rarely	Always	Usually	Rarely	Never	Always	Never	Never	Never	Never	Never	Never	Never	14
251	Always	Never	Sometimes	Rarely	Sometimes	Never	Rarely	Never	Never	Sometimes	Always	Sometimes	Sometimes	Always	Rarely	Rarely	Never	Rarely	Sometimes	Rarely	Sometimes	Never	Never	Never	Sometimes	Never	17
252	Never	Never	Always	Sometimes	Rarely	Never	Never	Sometimes	Never	Never	Never	Never	Usually	Rarely	Sometimes	Sometimes	Rarely	Always	Sometimes	Sometimes	Usually	Sometimes	Never	Never	Sometimes	Never	18
253	Usually	Never	Always	Sometimes	Always	Never	Never	Rarely	Never	Never	Sometimes	Never	Never	Usually	Rarely	Rarely	Sometimes	Sometimes	Always	Never	Never	Never	Never	Never	Sometimes	Never	18
254	Sometimes	Never	Sometimes	Never	Usually	Rarely	Rarely	Sometimes	Never	Sometimes	Sometimes	Sometimes	Never	Usually	Sometimes	Sometimes	Rarely	Never	Usually	Sometimes	Sometimes	Never	Never	Never	Sometimes	Rarely	17
255	Never	Never	Usually	Never	Sometimes	Never	Never	Always	Never	Rarely	Never	Never	Always	Never	Never	Never	Never	Rarely	Usually	Always	Sometimes	Never	Never	Never	Never	Never	15
256	Sometimes	Rarely	Usually	Sometimes	Usually	Rarely	Sometimes	Sometimes	Never	Never	Rarely	Rarely	Usually	Sometimes	Rarely	Rarely	Rarely	Usually	Sometimes	Sometimes	Sometimes	Rarely	Never	Rarely	Sometimes	Never	17
257	Usually	Sometimes	Usually	Rarely	Usually	Sometimes	Never	Never	Never	Rarely	Sometimes	Usually	Never	Usually	Rarely	Sometimes	Rarely	Never	Usually	Never	Sometimes	Rarely	Sometimes	Never	Rarely	Never	18
258	Sometimes	Sometimes	Usually	Rarely	Rarely	Never	Sometimes	Sometimes	Never	Sometimes	Rarely	Sometimes	Sometimes	Sometimes	Sometimes	Usually	Sometimes	Sometimes	Usually	Sometimes	Sometimes	Rarely	Rarely	Rarely	Sometimes	Never	20
259	Never	Never	Usually	Sometimes	Usually	Never	Never	Never	Rarely	Never	Never	Never	Sometimes	Never	Rarely	Usually	Never	Usually	Usually	Never	Sometimes	Never	Never	Never	Sometimes	Never	14
260	Usually	Rarely	Sometimes	Sometimes	Usually	Rarely	Rarely	Never	Never	Never	Sometimes	Sometimes	Sometimes	Rarely	Usually	Rarely	Never	Rarely	Usually	Never	Usually	Rarely	Never	Never	Sometimes	Never	16
261	Usually	Never	Always	Usually	Never	Never	Never	Never	Never	Sometimes	Usually	Never	Never	Sometimes	Sometimes	Never	Sometimes	Sometimes	Rarely	Never	Never	Sometimes	Never	Never	Never	Never	15
262	Sometimes	Rarely	Always	Never	Never	Never	Rarely	Never	Never	Rarely	Sometimes	Rarely	Rarely	Sometimes	Usually	Usually	Sometimes	Sometimes	Usually	Never	Sometimes	Rarely	Sometimes	Never	Rarely	Never	16
263	Sometimes	Never	Sometimes	Always	Rarely	Never	Rarely	Never	Never	Sometimes	Sometimes	Usually	Rarely	Never	Never	Rarely	Never	Sometimes	Sometimes	Never	Usually	Sometimes	Never	Never	Usually	Never	16
264	Never	Never	Usually	Never	Sometimes	Usually	Never	Never	Never	Never	Never	Sometimes	Never	Never	Rarely	Usually	Usually	Sometimes	Usually	Sometimes	Sometimes	Rarely	Never	Never	Rarely	Never	15
265	Never	Never	Usually	Sometimes	Sometimes	Never	Never	Never	Never	Sometimes	Never	Never	Usually	Rarely	Usually	Sometimes	Sometimes	Never	Usually	Never	Rarely	Never	Never	Never	Usually	Never	15
266	Always	Sometimes	Rarely	Sometimes	Sometimes	Never	Never	Always	Never	Rarely	Sometimes	Sometimes	Rarely	Never	Sometimes	Never	Never	Sometimes	Sometimes	Usually	Rarely	Sometimes	Never	Never	Never	Never	17
267	Never	Never	Always	Never	Sometimes	Never	Rarely	Sometimes	Never	Never	Never	Never	Usually	Never	Rarely	Rarely	Never	Sometimes	Usually	Sometimes	Rarely	Usually	Never	Never	Sometimes	Never	14
268	Sometimes	Rarely	Usually	Sometimes	Sometimes	Sometimes	Sometimes	Rarely	Never	Rarely	Rarely	Usually	Never	Sometimes	Rarely	Usually	Never	Rarely	Always	Rarely	Rarely	Rarely	Rarely	Rarely	Rarely	Never	15
269	Sometimes	Rarely	Sometimes	Usually	Always	Never	Never	Sometimes	Never	Sometimes	Sometimes	Never	Sometimes	Rarely	Sometimes	Rarely	Rarely	Sometimes	Usually	Rarely	Rarely	Sometimes	Never	Never	Sometimes	Rarely	17

270	Usually	Sometimes	Usually	Rarely	Never	Never	Sometimes	Usually	Never	Rarely	Sometimes	Sometimes	Sometimes	Sometimes	Sometimes	Sometimes	Sometimes	Usually	Sometimes	Sometimes	Sometimes	Never	Rarely	Sometimes	Sometimes	Never	22
271	Sometimes	Never	Usually	Sometimes	Never	Sometimes	Rarely	Never	Never	Rarely	Never	Sometimes	Rarely	Sometimes	Never	Sometimes	Usually	Never	Sometimes	Never	Sometimes	Sometimes	Sometimes	Never	Sometimes	Never	15
272	Rarely	Never	Always	Never	Rarely	Sometimes	Usually	Sometimes	Never	Never	Never	Never	Sometimes	Sometimes	Sometimes	Sometimes	Rarely	Never	Usually	Sometimes	Sometimes	Sometimes	Never	Never	Sometimes	Sometimes	18
273	Sometimes	Rarely	Usually	Sometimes	Rarely	Sometimes	Never	Never	Rarely	Rarely	Sometimes	Rarely	Never	Never	Sometimes	Sometimes	Rarely	Usually	Sometimes	Never	Usually	Never	Rarely	Rarely	Rarely	Usually	15
274	Usually	Sometimes	Always	Never	Never	Sometimes	Sometimes	Never	Never	Rarely	Usually	Sometimes	Never	Sometimes	Sometimes	Sometimes	Sometimes	Rarely	Usually	Never	Rarely	Rarely	Sometimes	Never	Sometimes	Never	19
275	Sometimes	Never	Always	Never	Sometimes	Sometimes	Never	Never	Never	Never	Never	Never	Never	Rarely	Sometimes	Never	Never	Always	Always	Never	Sometimes	Never	Never	Never	Sometimes	Never	15
276	Never	Never	Sometimes	Never	Sometimes	Never	Rarely	Usually	Never	Never	Sometimes	Never	Usually	Rarely	Always	Never	Never	Never	Usually	Sometimes	Rarely	Never	Never	Sometimes	Sometimes	Never	15
277	Always	Rarely	Rarely	Always	Rarely	Never	Never	Rarely	Never	Sometimes	Usually	Sometimes	Never	Sometimes	Sometimes	Never	Never	Usually	Rarely	Rarely	Sometimes	Rarely	Rarely	Rarely	Sometimes	Never	16
278	Never	Never	Usually	Never	Rarely	Never	Never	Usually	Never	Never	Never	Never	Usually	Never	Rarely	Usually	Rarely	Rarely	Always	Rarely	Sometimes	Sometimes	Never	Never	Sometimes	Rarely	14
279	Rarely	Rarely	Sometimes	Never	Rarely	Never	Always	Rarely	Never	Rarely	Never	Never	Sometimes	Rarely	Sometimes	Always	Sometimes	Usually	Usually	Rarely	Rarely	Sometimes	Never	Rarely	Sometimes	Never	16
280	Never	Never	Usually	Never	Always	Never	Never	Never	Rarely	Never	Never	Never	Sometimes	Never	Sometimes	Never	Never	Never	Always	Never	Sometimes	Never	Never	Never	Always	Rarely	14
281	Usually	Never	Usually	Never	Never	Rarely	Never	Rarely	Never	Never	Never	Never	Sometimes	Sometimes	Always	Never	Never	Never	Always	Never	Never	Never	Never	Never	Never	Never	12
282	Always	Never	Always	Never	Rarely	Never	Rarely	Never	Never	Rarely	Sometimes	Rarely	Never	Sometimes	Rarely	Usually	Never	Never	Usually	Never	Sometimes	Sometimes	Sometimes	Never	Sometimes	Never	16
283	Never	Never	Sometimes	Never	Always	Never	Sometimes	Sometimes	Never	Never	Never	Never	Sometimes	Rarely	Always	Sometimes	Never	Never	Always	Rarely	Sometimes	Rarely	Never	Never	Never	Never	15
284	Usually	Never	Usually	Usually	Sometimes	Never	Never	Never	Never	Sometimes	Sometimes	Never	Never	Never	Never	Never	Never	Never	Sometimes	Never	Sometimes	Never	Sometimes	Never	Never	Never	12
285	Never	Never	Sometimes	Never	Usually	Rarely	Rarely	Sometimes	Never	Never	Never	Never	Usually	Never	Sometimes	Never	Never	Never	Always	Rarely	Sometimes	Rarely	Never	Never	Usually	Never	13
286	Never	Never	Usually	Rarely	Sometimes	Sometimes	Sometimes	Sometimes	Never	Never	Rarely	Rarely	Sometimes	Usually	Rarely	Usually	Rarely	Never	Always	Rarely	Rarely	Rarely	Never	Never	Never	Never	14
287	Rarely	Never	Usually	Never	Usually	Never	Sometimes	Sometimes	Never	Sometimes	Sometimes	Sometimes	Sometimes	Rarely	Sometimes	Usually	Rarely	Rarely	Usually	Sometimes	Rarely	Rarely	Never	Never	Sometimes	Never	17
288	Never	Never	Always	Never	Always	Never	Never	Never	Never	Never	Sometimes	Never	Sometimes	Never	Sometimes	Sometimes	Rarely	Never	Usually	Rarely	Usually	Never	Never	Never	Sometimes	Never	15
289	Never	Never	Never	Never	Sometimes	Never	Never	Never	Never	Never	Never	Never	Always	Never	Never	Never	Never	Never	Always	Never	Always	Never	Never	Never	Always	Never	13
290	Never	Never	Usually	Never	Rarely	Always	Never	Sometimes	Never	Never	Never	Never	Sometimes	Sometimes	Sometimes	Rarely	Never	Sometimes	Sometimes	Never	Usually	Never	Never	Never	Sometimes	Never	14
291	Sometimes	Sometimes	Always	Never	Never	Sometimes	Never	Always	Never	Never	Never	Never	Sometimes	Never	Never	Never	Never	Never	Always	Sometimes	Sometimes	Never	Never	Never	Sometimes	Sometimes	17
292	Usually	Never	Always	Never	Sometimes	Rarely	Sometimes	Never	Never	Never	Sometimes	Sometimes	Never	Always	Never	Never	Never	Sometimes	Usually	Never	Never	Sometimes	Sometimes	Never	Never	Never	17
293	Rarely	Never	Sometimes	Sometimes	Sometimes	Sometimes	Rarely	Rarely	Never	Sometimes	Sometimes	Sometimes	Usually	Rarely	Sometimes	Rarely	Some	Sometimes	Sometimes	Sometimes	Usually	Usually	Sometimes	Never	Sometimes	Sometimes	21

																	times										
294	Sometimes	Never	Always	Never	Usually	Rarely	Never	Never	Never	Never	Never	Never	Never	Never	Sometimes	Never	Never	Sometimes	Always	Never	Never	Never	Never	Never	Usually	Never	13
295	Never	Never	Always	Never	Never	Never	Never	Sometimes	Never	Never	Never	Never	Sometimes	Never	Usually	Sometimes	Rarely	Rarely	Usually	Sometimes	Rarely	Never	Never	Never	Sometimes	Never	12
296	Never	Rarely	Sometimes	Never	Sometimes	Never	Rarely	Never	Never	Never	Never	Sometimes	Never	Rarely	Sometimes	Usually	Never	Always	Usually	Never	Rarely	Sometimes	Never	Never	Rarely	Rarely	12
297	Rarely	Rarely	Always	Rarely	Sometimes	Sometimes	Sometimes	Rarely	Rarely	Never	Sometimes	Sometimes	Never	Rarely	Rarely	Usually	Sometimes	Never	Always	Never	Rarely	Sometimes	Sometimes	Rarely	Rarely	Rarely	16
298	Never	Never	Always	Never	Never	Sometimes	Rarely	Sometimes	Never	Never	Never	Sometimes	Sometimes	Sometimes	Rarely	Never	Never	Usually	Always	Rarely	Sometimes	Never	Never	Never	Sometimes	Never	15
299	Sometimes	Sometimes	Sometimes	Sometimes	Sometimes	Sometimes	Sometimes	Sometimes	Never	Sometimes	Sometimes	Sometimes	Never	Sometimes	Sometimes	Sometimes	Rarely	Sometimes	Sometimes	Sometimes	Sometimes	Sometimes	Usually	Rarely	Rarely	Never	21
300	Sometimes	Never	Always	Never	Usually	Usually	Sometimes	Never	Never	Sometimes	Sometimes	Usually	Never	Sometimes	Sometimes	Rarely	Never	Sometimes	Sometimes	Sometimes	Sometimes	Sometimes	Never	Never	Sometimes	Never	21
301	Never	Rarely	Usually	Sometimes	Sometimes	Rarely	Rarely	Never	Never	Never	Never	Never	Sometimes	Never	Always	Sometimes	Rarely	Sometimes	Usually	Sometimes	Rarely	Sometimes	Never	Rarely	Rarely	Never	14
302	Usually	Rarely	Usually	Sometimes	Sometimes	Sometimes	Sometimes	Never	Never	Rarely	Rarely	Sometimes	Sometimes	Sometimes	Usually	Sometimes	Rarely	Sometimes	Usually	Never	Never	Never	Never	Never	Rarely	Never	17
303	Sometimes	Rarely	Usually	Rarely	Rarely	Rarely	Always	Never	Never	Rarely	Rarely	Rarely	Never	Rarely	Rarely	Usually	Never	Never	Usually	Never	Never	Usually	Never	Rarely	Rarely	Never	12
304	Sometimes	Sometimes	Always	Rarely	Sometimes	Rarely	Never	Never	Never	Never	Never	Rarely	Sometimes	Rarely	Sometimes	Rarely	Never	Usually	Sometimes	Rarely	Sometimes	Usually	Never	Never	Sometimes	Never	15
305	Never	Never	Rarely	Never	Never	Usually	Never	Never	Never	Never	Never	Never	Never	Sometimes	Rarely	Usually	Usually	Never	Always	Never	Never	Sometimes	Never	Never	Sometimes	Never	12
306	Sometimes	Never	Always	Rarely	Sometimes	Never	Rarely	Rarely	Never	Rarely	Sometimes	Rarely	Rarely	Sometimes	Sometimes	Sometimes	Rarely	Sometimes	Usually	Sometimes	Sometimes	Rarely	Never	Rarely	Sometimes	Never	15
307	Never	Sometimes	Always	Never	Sometimes	Rarely	Never	Never	Never	Never	Never	Never	Usually	Rarely	Sometimes	Rarely	Rarely	Usually	Usually	Sometimes	Rarely	Sometimes	Never	Never	Never	Never	14
308	Always	Rarely	Rarely	Sometimes	Sometimes	Never	Never	Never	Never	Rarely	Usually	Never	Never	Usually	Sometimes	Sometimes	Rarely	Never	Usually	Never	Sometimes	Never	Never	Never	Never	Never	14
309	Never	Never	Never	Never	Never	Never	Never	Usually	Never	Never	Sometimes	Sometimes	Sometimes	Sometimes	Never	Never	Never	Never	Always	Sometimes	Sometimes	Never	Never	Never	Never	Never	11
310	Sometimes	Never	Usually	Rarely	Usually	Sometimes	Rarely	Rarely	Never	Never	Sometimes	Rarely	Never	Rarely	Sometimes	Rarely	Never	Sometimes	Usually	Sometimes	Sometimes	Sometimes	Sometimes	Sometimes	Sometimes	Never	17
311	Never	Never	Usually	Never	Never	Usually	Never	Never	Never	Never	Never	Never	Never	Rarely	Never	Usually	Sometimes	Never	Usually	Never	Sometimes	Never	Never	Never	Sometimes	Never	11
312	Sometimes	Rarely	Usually	Sometimes	Usually	Sometimes	Rarely	Sometimes	Never	Sometimes	Sometimes	Sometimes	Rarely	Rarely	Sometimes	Rarely	Rarely	Sometimes	Usually	Never	Rarely	Sometimes	Never	Never	Sometimes	Never	17
313	Rarely	Never	Always	Never	Sometimes	Never	Rarely	Never	Never	Never	Never	Never	Never	Never	Rarely	Never	Always	Never	Always	Never	Rarely	Never	Never	Never	Sometimes	Sometimes	12
314	Sometimes	Rarely	Sometimes	Sometimes	Rarely	Never	Sometimes	Never	Never	Rarely	Sometimes	Sometimes	Never	Usually	Rarely	Sometimes	Sometimes	Never	Usually	Never	Rarely	Sometimes	Sometimes	Never	Sometimes	Never	15
315	Sometimes	Sometimes	Usually	Never	Sometimes	Sometimes	Rarely	Never	Never	Never	Never	Sometimes	Never	Sometimes	Never	Never	Rarely	Sometimes	Always	Rarely	Sometimes	Never	Rarely	Never	Sometimes	Never	14
316	Never	Never	Usually	Rarely	Sometimes	Sometimes	Rarely	Rarely	Never	Never	Never	Never	Rarely	Rarely	Always	Usually	Never	Sometimes	Sometimes	Never	Rarely	Sometimes	Never	Sometimes	Sometimes	Never	14

317	Sometimes	Never	Usually	Never	Rarely	Never	Sometimes	Usually	Never	Never	Never	Never	Rarely	Rarely	Sometimes	Sometimes	Never	Rarely	Always	Never	Rarely	Sometimes	Sometimes	Sometimes	Rarely	Never	14
318	Sometimes	Never	Always	Rarely	Usually	Rarely	Sometimes	Rarely	Never	Never	Sometimes	Rarely	Never	Rarely	Rarely	Sometimes	Rarely	Sometimes	Usually	Rarely	Rarely	Never	Rarely	Never	Sometimes	Never	13
319	Never	Never	Usually	Sometimes	Rarely	Rarely	Rarely	Rarely	Never	Never	Rarely	Never	Never	Never	Rarely	Usually	Never	Never	Usually	Never	Rarely	Usually	Rarely	Sometimes	Sometimes	Never	11
320	Sometimes	Never	Always	Rarely	Rarely	Never	Never	Never	Rarely	Never	Rarely	Never	Sometimes	Rarely	Never	Never	Rarely	Always	Sometimes	Sometimes	Never	Never	Never	Never	Usually	Never	12
321	Rarely	Never	Usually	Usually	Usually	Never	Rarely	Never	Never	Never	Never	Never	Sometimes	Never	Sometimes	Rarely	Never	Never	Sometimes	Never	Sometimes	Sometimes	Never	Sometimes	Usually	Never	14
322	Rarely	Rarely	Usually	Never	Rarely	Usually	Usually	Sometimes	Never	Rarely	Rarely	Rarely	Sometimes	Rarely	Rarely	Usually	Never	Never	Usually	Rarely	Never	Never	Sometimes	Rarely	Rarely	Never	13
323	Rarely	Sometimes	Usually	Never	Rarely	Never	Rarely	Sometimes	Never	Never	Never	Never	Sometimes	Rarely	Sometimes	Sometimes	Sometimes	Sometimes	Usually	Sometimes	Sometimes	Never	Never	Never	Sometimes	Never	14
324	Sometimes	Rarely	Usually	Sometimes	Rarely	Usually	Sometimes	Rarely	Never	Never	Sometimes	Sometimes	Sometimes	Sometimes	Rarely	Rarely	Never	Sometimes	Sometimes	Rarely	Sometimes	Never	Rarely	Never	Rarely	Never	14
325	Sometimes	Sometimes	Usually	Usually	Rarely	Sometimes	Sometimes	Never	Never	Sometimes	Sometimes	Sometimes	Rarely	Sometimes	Rarely	Rarely	Rarely	Never	Sometimes	Rarely	Sometimes	Rarely	Sometimes	Never	Sometimes	Never	16
326	Never	Never	Sometimes	Rarely	Sometimes	Never	Rarely	Rarely	Never	Rarely	Never	Never	Always	Rarely	Rarely	Sometimes	Rarely	Usually	Usually	Sometimes	Sometimes	Sometimes	Never	Never	Sometimes	Never	14
327	Sometimes	Sometimes	Usually	Sometimes	Never	Never	Never	Never	Never	Sometimes	Rarely	Rarely	Sometimes	Usually	Rarely	Rarely	Never	Usually	Sometimes	Rarely	Sometimes	Rarely	Never	Rarely	Usually	Never	15
328	Rarely	Never	Usually	Sometimes	Never	Never	Never	Never	Never	Sometimes	Never	Usually	Sometimes	Never	Never	Never	Never	Never	Usually	Never	Sometimes	Never	Never	Never	Always	Never	13
329	Sometimes	Never	Sometimes	Sometimes	Never	Sometimes	Never	Never	Never	Sometimes	Usually	Never	Never	Sometimes	Never	Sometimes	Usually	Never	Rarely	Never	Rarely	Sometimes	Sometimes	Never	Rarely	Never	13
330	Sometimes	Sometimes	Sometimes	Never	Usually	Rarely	Rarely	Rarely	Never	Never	Sometimes	Sometimes	Never	Rarely	Usually	Rarely	Rarely	Rarely	Usually	Never	Rarely	Never	Never	Never	Sometimes	Never	12
331	Rarely	Never	Usually	Never	Usually	Rarely	Rarely	Never	Never	Rarely	Rarely	Sometimes	Sometimes	Sometimes	Never	Rarely	Never	Never	Usually	Never	Rarely	Rarely	Never	Sometimes	Sometimes	Never	11
332	Usually	Never	Usually	Never	Rarely	Rarely	Sometimes	Rarely	Never	Rarely	Never	Never	Never	Never	Sometimes	Rarely	Never	Usually	Always	Never	Never	Rarely	Never	Never	Never	Rarely	11
333	Sometimes	Rarely	Usually	Never	Always	Never	Never	Rarely	Never	Never	Rarely	Never	Sometimes	Never	Sometimes	Rarely	Never	Never	Always	Rarely	Sometimes	Never	Never	Never	Rarely	Never	12
334	Rarely	Rarely	Sometimes	Rarely	Sometimes	Sometimes	Sometimes	Usually	Never	Never	Never	Rarely	Sometimes	Sometimes	Sometimes	Sometimes	Rarely	Never	Usually	Sometimes	Rarely	Sometimes	Never	Sometimes	Rarely	Never	15
335	Never	Never	Usually	Sometimes	Sometimes	Never	Never	Never	Never	Never	Rarely	Rarely	Sometimes	Rarely	Sometimes	Never	Never	Rarely	Sometimes	Rarely	Sometimes	Sometimes	Never	Never	Usually	Never	11
336	Never	Never	Rarely	Never	Never	Never	Never	Never	Never	Never	Never	Never	Always	Never	Never	Never	Sometimes	Never	Usually	Never	Sometimes	Never	Never	Never	Always	Never	10
337	Sometimes	Rarely	Usually	Sometimes	Rarely	Never	Rarely	Sometimes	Never	Never	Never	Never	Usually	Rarely	Rarely	Rarely	Never	Usually	Sometimes	Rarely	Rarely	Never	Never	Never	Sometimes	Never	11
338	Never	Never	Usually	Never	Never	Sometimes	Rarely	Never	Never	Never	Never	Never	Usually	Never	Rarely	Rarely	Rarely	Usually	Usually	Never	Never	Never	Never	Never	Rarely	Never	9
339	Sometimes	Sometimes	Usually	Sometimes	Sometimes	Usually	Sometimes	Sometimes	Never	Never	Never	Sometimes	Rarely	Never	Rarely	Never	Never	Never	Usually	Rarely	Rarely	Never	Rarely	Never	Sometimes	Never	14
340	Rarely	Sometimes	Usually	Sometimes	Sometimes	Never	Rarely	Rarely	Never	Never	Rarely	Sometimes	Sometimes	Sometimes	Sometimes	Rarely	Sometimes	Sometimes	Always	Rarely	Rarely	Rarely	Rarely	Never	Rarely	Never	14

341	Sometimes	Sometimes	Always	Sometimes	Never	Never	Rarely	Never	Never	Never	Sometimes	Never	Never	Never	Never	Never	Never	Never	Sometimes	Never	Never	Never	Sometimes	Never	Sometimes	Never	10
342	Rarely	Rarely	Usually	Never	Rarely	Never	Rarely	Never	Never	Never	Never	Never	Rarely	Rarely	Sometimes	Never	Rarely	Usually	Usually	Sometimes	Sometimes	Rarely	Never	Never	Sometimes	Never	10
343	Never	Never	Usually	Never	Never	Sometimes	Never	Never	Never	Rarely	Never	Sometimes	Sometimes	Rarely	Never	Sometimes	Never	Usually	Usually	Sometimes	Rarely	Never	Never	Never	Rarely	Never	11
344	Sometimes	Rarely	Usually	Rarely	Sometimes	Sometimes	Sometimes	Sometimes	Never	Never	Rarely	Rarely	Sometimes	Sometimes	Rarely	Sometimes	Never	Never	Always	Rarely	Rarely	Rarely	Never	Never	Sometimes	Never	14
345	Never	Never	Sometimes	Never	Never	Never	Never	Sometimes	Never	Never	Never	Never	Always	Never	Sometimes	Rarely	Never	Never	Usually	Never	Sometimes	Never	Never	Never	Sometimes	Never	10
346	Usually	Sometimes	Sometimes	Sometimes	Sometimes	Never	Rarely	Never	Never	Never	Sometimes	Sometimes	Rarely	Rarely	Rarely	Rarely	Never	Never	Usually	Never	Never	Never	Rarely	Rarely	Sometimes	Rarely	11
347	Sometimes	Rarely	Sometimes	Never	Always	Rarely	Rarely	Sometimes	Never	Never	Sometimes	Never	Never	Never	Never	Rarely	Never	Never	Always	Never	Never	Never	Sometimes	Sometimes	Never	Never	12
348	Usually	Never	Usually	Rarely	Sometimes	Sometimes	Sometimes	Never	Never	Never	Rarely	Rarely	Never	Sometimes	Never	Rarely	Never	Rarely	Sometimes	Never	Never	Never	Sometimes	Never	Usually	Never	12
349	Never	Never	Sometimes	Sometimes	Never	Never	Never	Sometimes	Never	Rarely	Never	Never	Sometimes	Never	Always	Never	Never	Never	Rarely	Rarely	Sometimes	Never	Never	Never	Never	Never	8
350	Sometimes	Never	Sometimes	Rarely	Sometimes	Sometimes	Never	Rarely	Never	Rarely	Sometimes	Rarely	Never	Sometimes	Rarely	Rarely	Never	Sometimes	Usually	Rarely	Sometimes	Rarely	Rarely	Rarely	Sometimes	Never	11
351	Sometimes	Never	Usually	Never	Sometimes	Never	Never	Never	Never	Never	Rarely	Sometimes	Never	Rarely	Rarely	Rarely	Never	Sometimes	Always	Rarely	Sometimes	Rarely	Rarely	Never	Rarely	Rarely	10
352	Sometimes	Rarely	Always	Sometimes	Sometimes	Never	Never	Never	Never	Never	Sometimes	Never	Rarely	Sometimes	Never	Sometimes	Never	Rarely	Never	Never	Usually	Rarely	Rarely	Rarely	Never	Never	11
353	Never	Never	Sometimes	Never	Never	Never	Never	Sometimes	Never	Never	Never	Never	Usually	Never	Never	Never	Never	Sometimes	Always	Never	Usually	Sometimes	Never	Never	Never	Never	11
354	Rarely	Sometimes	Always	Sometimes	Rarely	Rarely	Sometimes	Never	Rarely	Rarely	Sometimes	Never	Never	Sometimes	Rarely	Rarely	Rarely	Sometimes	Sometimes	Never	Sometimes	Sometimes	Sometimes	Never	Sometimes	Rarely	14
355	Sometimes	Rarely	Sometimes	Never	Sometimes	Never	Never	Sometimes	Sometimes	Rarely	Sometimes	Rarely	Never	Sometimes	Sometimes	Never	Never	Never	Sometimes	Rarely	Sometimes	Never	Never	Rarely	Sometimes	Sometimes	12
356	Rarely	Never	Sometimes	Never	Always	Rarely	Never	Never	Never	Never	Never	Never	Rarely	Never	Rarely	Sometimes	Rarely	Never	Usually	Never	Rarely	Never	Rarely	Never	Sometimes	Never	8
357	Usually	Sometimes	Usually	Sometimes	Rarely	Sometimes	Sometimes	Never	Never	Rarely	Never	Sometimes	Never	Sometimes	Never	Sometimes	Never	Never	Usually	Never	Rarely	Rarely	Rarely	Never	Rarely	Never	13
358	Sometimes	Never	Sometimes	Usually	Never	Never	Never	Never	Never	Rarely	Rarely	Always	Rarely	Never	Never	Never	Never	Sometimes	Never	Never	Never	Never	Never	Never	Never	Never	8
359	Sometimes	Sometimes	Sometimes	Never	Rarely	Never	Sometimes	Rarely	Never	Rarely	Sometimes	Sometimes	Rarely	Sometimes	Sometimes	Sometimes	Never	Never	Usually	Never	Rarely	Rarely	Sometimes	Sometimes	Sometimes	Never	14
360	Usually	Never	Usually	Never	Never	Never	Never	Never	Never	Never	Never	Never	Never	Rarely	Never	Never	Never	Never	Always	Never	Never	Never	Never	Never	Never	Never	7
361	Never	Never	Sometimes	Never	Sometimes	Never	Never	Sometimes	Never	Never	Never	Never	Always	Never	Sometimes	Never	Never	Never	Sometimes	Never	Sometimes	Never	Never	Never	Never	Never	9
362	Rarely	Never	Rarely	Never	Sometimes	Sometimes	Sometimes	Never	Never	Never	Rarely	Never	Never	Rarely	Rarely	Always	Never	Never	Always	Never	Never	Never	Rarely	Never	Never	Never	9
363	Sometimes	Rarely	Rarely	Rarely	Never	Rarely	Sometimes	Sometimes	Never	Never	Never	Never	Never	Rarely	Sometimes	Rarely	Rarely	Never	Always	Never	Sometimes	Never	Never	Never	Sometimes	Never	9
364	Sometimes	Never	Usually	Rarely	Rarely	Sometimes	Sometimes	Never	Never	Sometimes	Sometimes	Sometimes	Never	Sometimes	Never	Sometimes	Sometimes	Usually	Sometimes	Never	Sometimes	Sometimes	Rarely	Never	Sometimes	Never	17

365	Rarely	Sometimes	Sometimes	Sometimes	Sometimes	Sometimes	Sometimes	Rarely	Never	Sometimes	Sometimes	Rarely	Rarely	Sometimes	Sometimes	Sometimes	Sometimes	Never	Sometimes	Sometimes	Sometimes	Sometimes	Sometimes	Rarely	Rarely	Never	17
366	Rarely	Never	Usually	Rarely	Always	Sometimes	Rarely	Sometimes	Never	Sometimes	Never	Never	Sometimes	Rarely	Never	Rarely	Rarely	Rarely	Usually	Sometimes	Sometimes	Never	Rarely	Never	Rarely	Never	13
367	Never	Never	Sometimes	Never	Sometimes	Never	Never	Sometimes	Never	Never	Never	Never	Always	Never	Rarely	Never	Never	Sometimes	Sometimes	Sometimes	Usually	Never	Never	Never	Rarely	Never	11
368	Sometimes	Never	Always	Sometimes	Rarely	Never	Sometimes	Never	Never	Never	Never	Rarely	Sometimes	Rarely	Sometimes	Sometimes	Sometimes	Sometimes	Sometimes	Rarely	Never	Sometimes	Never	Never	Rarely	Never	13
369	Rarely	Never	Never	Rarely	Never	Never	Never	Never	Never	Never	Never	Never	Never	Never	Always	Never	Never	Never	Always	Never	Never	Never	Never	Never	Sometimes	Never	7
370	Rarely	Rarely	Sometimes	Rarely	Sometimes	Usually	Rarely	Rarely	Never	Rarely	Sometimes	Sometimes	Rarely	Sometimes	Rarely	Sometimes	Rarely	Never	Sometimes	Rarely	Rarely	Never	Sometimes	Never	Sometimes	Never	11
371	Usually	Rarely	Usually	Rarely	Rarely	Never	Never	Never	Never	Sometimes	Rarely	Never	Never	Rarely	Never	Never	Never	Never	Sometimes	Never	Never	Never	Rarely	Never	Sometimes	Never	7
372	Usually	Never	Sometimes	Rarely	Sometimes	Never	Never	Never	Never	Never	Never	Never	Sometimes	Never	Never	Never	Never	Never	Usually	Never	Never	Never	Never	Never	Usually	Never	9
373	Never	Never	Always	Never	Never	Never	Never	Never	Never	Never	Never	Never	Never	Never	Never	Sometimes	Never	Never	Always	Never	Never	Never	Never	Never	Sometimes	Never	8
374	Sometimes	Never	Sometimes	Sometimes	Rarely	Never	Rarely	Never	Never	Never	Rarely	Never	Rarely	Rarely	Never	Sometimes	Never	Sometimes	Sometimes	Sometimes	Usually	Rarely	Never	Never	Sometimes	Never	10
375	Sometimes	Rarely	Sometimes	Sometimes	Usually	Rarely	Never	Never	Never	Never	Rarely	Rarely	Rarely	Never	Usually	Rarely	Never	Never	Sometimes	Never	Rarely	Sometimes	Never	Never	Never	Never	9
376	Rarely	Never	Usually	Rarely	Rarely	Never	Never	Never	Sometimes	Never	Rarely	Never	Sometimes	Rarely	Never	Rarely	Never	Rarely	Usually	Rarely	Usually	Never	Never	Never	Rarely	Sometimes	9
377	Rarely	Rarely	Sometimes	Rarely	Sometimes	Sometimes	Sometimes	Never	Never	Never	Never	Never	Usually	Sometimes	Sometimes	Sometimes	Never	Never	Sometimes	Never	Rarely	Sometimes	Never	Rarely	Rarely	Never	11
378	Never	Never	Never	Never	Never	Never	Never	Never	Never	Never	Never	Never	Sometimes	Never	Rarely	Never	Never	Never	Always	Never	Never	Never	Never	Never	Sometimes	Never	5
379	Sometimes	Rarely	Rarely	Sometimes	Sometimes	Sometimes	Sometimes	Never	Never	Never	Sometimes	Never	Never	Never	Never	Rarely	Never	Never	Rarely	Never	Never	Never	Rarely	Never	Rarely	Never	6
380	Sometimes	Rarely	Sometimes	Rarely	Sometimes	Rarely	Rarely	Sometimes	Never	Never	Sometimes	Rarely	Rarely	Sometimes	Sometimes	Sometimes	Rarely	Rarely	Rarely	Rarely	Sometimes	Sometimes	Rarely	Rarely	Rarely	Never	10
381	Sometimes	Never	Usually	Sometimes	Never	Never	Never	Sometimes	Never	Rarely	Sometimes	Never	Rarely	Sometimes	Never	Rarely	Never	Sometimes	Sometimes	Never	Rarely	Never	Never	Never	Rarely	Never	9
382	Rarely	Never	Usually	Never	Sometimes	Never	Never	Sometimes	Never	Rarely	Never	Never	Never	Never	Rarely	Rarely	Never	Rarely	Sometimes	Rarely	Sometimes	Sometimes	Never	Never	Sometimes	Never	8
383	Never	Never	Never	Sometimes	Sometimes	Never	Never	Never	Never	Never	Never	Never	Sometimes	Rarely	Rarely	Rarely	Never	Never	Usually	Never	Rarely	Never	Never	Rarely	Sometimes	Never	6
384	Never	Never	Usually	Never	Never	Never	Never	Never	Never	Never	Never	Never	Never	Never	Never	Never	Never	Never	Never	Never	Never	Never	Never	Never	Never	Never	2
385	Sometimes	Sometimes	Rarely	Rarely	Rarely	Never	Never	Never	Never	Never	Rarely	Never	Never	Never	Never	Never	Never	Never	Sometimes	Never	Rarely	Never	Never	Never	Sometimes	Never	4
